# Optimal collective decision making: consensus, accuracy and the effects of limited access to information

**DOI:** 10.1038/s41598-020-73853-z

**Published:** 2020-10-12

**Authors:** Evelin Berekméri, Anna Zafeiris

**Affiliations:** 1 Department of Biological Physics, Eötvös University, Budapest, 1117 Hungary; 2 MTA-ELTE ‘Lendūlet’ Collective Behaviour Research Group, Hungarian Academy of Sciences, Eötvös University, Budapest, 1117 Hungary; 3 MTA-ELTE Statistical and Biological Physics Research Group, Hungarian Academy of Sciences, Budapest, 1117 Hungary

**Keywords:** Complex networks, Biological physics

## Abstract

“Knowledge is power”—holds the popular proverb, because knowledge and information is indeed one of the cornerstones of effective decision making, a requisite all living beings face continually. In fact, effective decision making is a matter of life and death, for individuals and groups alike. Furthermore, in case of group decisions, *consensus* is also often desirable. This latter one has been studied extensively by means of formal (mathematical) tools (in the field of opinion dynamics), while the first requirement, the process of *yielding accurate information* has been largely neglected, at least so far. In the present paper we study the *optimal* structure of groups which are embedded into an external, observable environment for (i) reaching consensus (ii) having well-informed members, and (iii) for those cases when both aspects are equally important. The groups are characterised by their communication networks and individual properties. We find that the group structures fundamentally differ from each other since having well-informed members requires highly specialised individuals embedded into a structured communication network, while consensus is promoted by non-hierarchical networks in which individuals participate equally. We also find that—contrary to intuition—*high* access to information calls forth hierarchy, and that suggestibility promotes accuracy, not consensus.

## Introduction

According to Nobel-laureate Daniel Kahneman, “Whatever else it produces, an organization is a factory that manufactures judgments and decisions”^1^. These decisions are of many kinds: they can relate to new investments, fundraising, change of profile, the hire of new employees, adaptation of new technologies, expansions to new areas—just to mention a few. What is common in these cases is that they are usually made by a few—top a most a few dozen^2^—decision makers, all of whom have only partial access to the information necessary to make well-founded decisions: for example, considering a company, one of the decision-makers might have detailed information regarding the law environment in the country they are considering new investment in; an other one might be familiar with the local education system and the quality of expertise; a third member might be informed regarding the conditions of the local market, etc. In other words, different individuals see different aspects, “facets” of the same problem, and only in the most simple cases do all decision-makers oversee all the aspects, the complete “*environment*”—an observable “external reality” which includes important information regarding the problem or situation to be decided about.

From a more general point of view, not only organizations, but all communities—animal and human alike—are “decision making factories”, since all of them face a constant pressure for making collective decisions^3–6^ (By “community” we mean a group with more or less stable membership performing common actions^7^). Moreover, the quality of these decisions are of fundamental importance, since often the very existence of the group depends on them: if an animal gang makes a mistake regarding the safety or position of a night-lair, it might easily be attacked by predators. If a flock of birds navigates incorrectly towards its winter location, it might easily lose its way and find itself in cold or nutrition-poor locations. If the decision-making board of a company takes a bad decision regarding a new investment, it might easily go bankrupt. In these (and many other real-life) cases the quality of the decisions fundamentally depend on the *accuracy and completeness* of information regarding the environment^8,9^. In short, complete and accurate information is a fundamental requirement for making good decisions^10,11^. Furthermore, in case of groups, *consensus* plays a central role as well, since it ensures cohesive, close-knit communities by warranting the feeling for the members that they create their future together (As a matter of fact, many considers consensus as *the* most important aspect of group decision making^12,13^, an opinion which seems to be supported by the vast amount of literature studying its dynamics and emergence^14–16^).

In the present paper we focus on the relation of these two aspects—consensus and well-informedness—in the form of studying and comparing the features of groups promoting one or the other aspect. The groups are described by their communication networks, and by the characteristics of the individuals: their communication activity, observation activity, and their level of suggestibility (features that will be discussed in details in “The model” section). We optimise these values—including the communication network—by means of genetic algorithm^17^, with three different “optimality” definitions:

(i) the first one considers a group “optimal”, if it ensures that the members possess accurate information regarding an observable external environment (to which individuals have only partial access to). We refer to this condition as being “*well-informed*”.
(ii) according to the second definition, a group is “optimal”, if its structure promotes fast emergence of consensus, and finally
(iii) the third one considers a group “optimal” if it promotes both requirements with equal strength.
  Importantly, as mentioned above, we also take into account the members’ limited access to information—a circumstance which, despite its trivial nature, rarely is incorporated in information-diffusion models^18,19^. This means, though tacitly, that individuals are assumed to have full access to the information needed to form the decision. As we have seen, this assumption holds only in simple cases, while in most real-life decisions the environment of the problem is more complex^20,21^.

We find that the features of the groups promoting consensus vs. well-informedness fundamentally differ from each other: *Consensus*—in accordance to management experts^22,23^—require a highly egalitarian group structure in which all members participate in the spread of information intensively and equally, with basically no specialization among the agents. However, in case individuals have good access to information, the optimal strategy to reach consensus is to complement intense communication with intense observation, despite the fact that in this case accuracy is not a requisite at all, and observation is associated with much higher costs than communication (referring to time, effort, material resources, etc.). This phenomenon most probably originates from the fact that reaching consensus, based on communication alone, is often extremely time-consuming^24^.

In contrast, having *well-informed* individuals requires specialisation of members with respect to their activity in circulating the information: some become very active, while others—the majority—cease to initiate communication. And what is more, this phenomenon gets more and more pronounced with the *growth* of the ratio of access to information. In other words, the *more* information decision-makers have access to, the *more* hierarchical the optimal communication network is, with more specialised members. Furthermore—also unexpectedly—we find that high suggestibility (conformity) values correspond to well-informed agents, but not consensus, to which it correlates only minimally. We shall discuss these results in detail in the “Results” section.

## The model

According to the literature, real-life consensus reaching and information sharing processes take place in a convergent multistage way: the decision-makers (who, at the beginning often have different views and information) communicate their individual opinions and beliefs while the consensus level is not considered to be satisfactorily high enough. During this process, agents bring their positions closer to each others’ standpoint by exchanging their information^12^.

In order to formulate this realistic and general description, with incorporating the very real-life but usually overlooked assumption that decision-makers normally have access only to a small portion of the information they need in order to make a well-founded decision, we have designed the following minimal-model: a group of *N* agents (decision makers) confront a problem with many facets, which they can oversee only with a joint effort. They check up and discuss data related to these various aspects in several rounds, a process during which they alter their views and information. The “complexity” of the problem (or “environment”, in which the decision has to be made) is tuned by a parameter *K* which can be interpreted as the number of “facets” or aspects that has to be known in order to make a well-founded decision; accordingly, higher values of *K* refer to more complex problems. More formally, the “environment” is represented by a *K*-long number-series, consisting of real values taken from the [0, 1] interval with uniform distribution (a choice which follows from the assumption that these environmental elements are independent from each other). Each agent has access only to a limited ratio of the environment-vector, set by a parameter *H*: if $H=0.1$, then individuals “see” 10% of the environment vector; if $H=0.5$, than they see half of it, while in case $H=1$, each agent has access to the entire environment vector. *H* is the same for all agents.

**Figure 1:**
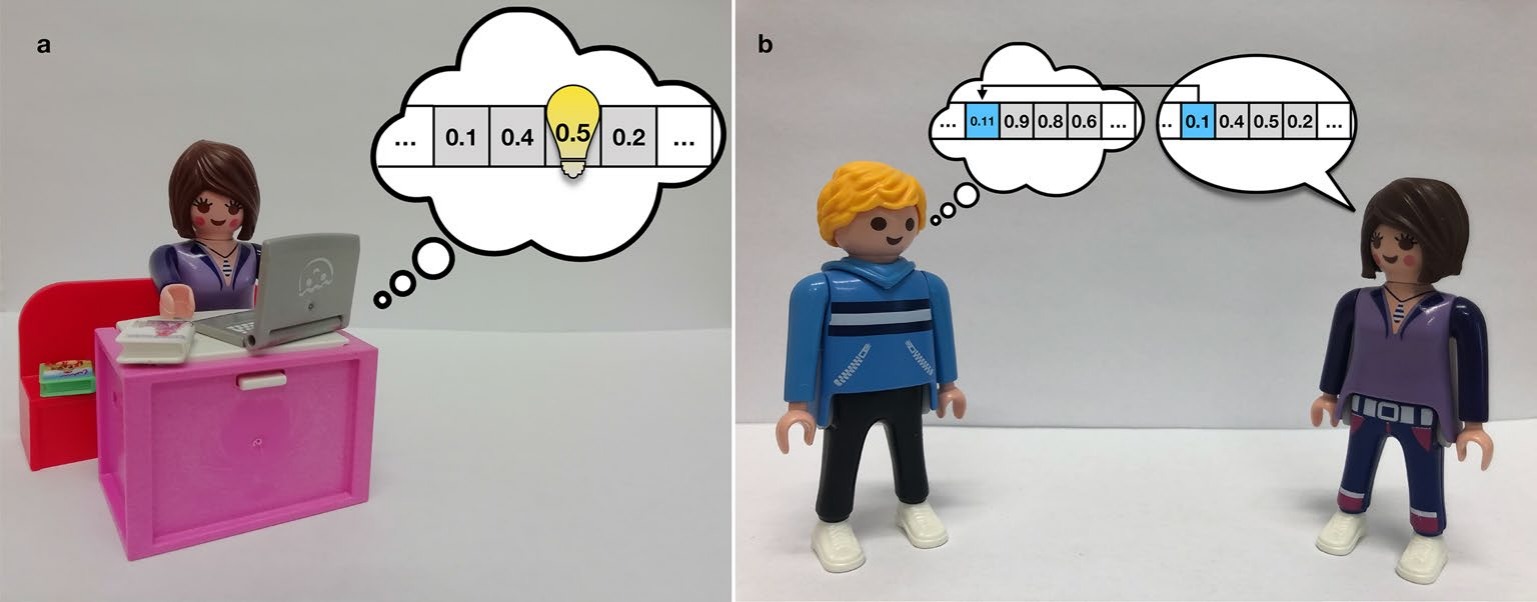
The main features of the model. Individuals might alter their beliefs either by *observation* (**a**) or by *communication* (**b**). Observation (**a**) is an activity during which agents retrace data from reliable sources personally. Importantly, individuals can not oversee the entire environment, only a portion of it, tuned by a parameter called *H* ($H \in [0, 1])$. Observation is costly (in terms of time, effort or other resources), but provides accurate information. In contrast, communication (**b**) is less costly, but, at the same time, provides less reliably information. The flow of information is one directional, and during communication the one who “talks” alters the beliefs (more precisely: a randomly chosen element of the belief-vector) of his/her conversation partner. ©PLAYMOBIL/ geobra Brandstätter Stiftung & Co. KG.

Each individual has an image of the environment, represented by a *K*-long sequence of real numbers; these are the “belief vectors”, which are set randomly at the beginning of each run. The elements of these vectors may alter due to two activities: (i) communication, or (ii) “observation”. The latter one, observation, refers to an activity during which individuals observe their environment directly: they look up relevant data, making personal researches or measurements (see Fig. 1a). This activity is costly, but produces precise information (Cost can refer to time, effort, or the usage of any other resources). In contrast, communication is less costly, but less reliable as well: if the individual *sending* the information happens to have accurate data regarding that certain element of the environment they are “talking” about (see Fig. 1b), then the accuracy level of the one receiving the information will increase, otherwise it will decrease. In any case, the “mind” of the receiver draws nearer to the mind of the sender, with a ratio proportional to the receiver’s suggestibility (More suggestible people alter their minds more easily).

Agents (decision makers) form groups which are defined by the following parameters:

- A communication network, represented by a weighted, directed network ${\mathbf {A}}=(a_{ij})\in {\mathbb {R}}^{N \times N}$. The $a_{ij}$ element of this matrix denotes the *probability of communication* (flow of information) between agents $i \rightarrow j$, *in case agent i chooses to communicate*. In this case, agent *i* modifies the “beliefs” (information) of agent *j*, but not vice versa (In order to track the flow of information more accurately, communication in this model is one directional, during which the “sender” influences the beliefs of the “receiver”). Since $a_{ij}$ is a probability, it takes values from the [0, 1] interval, furthermore $a_{ii}=0$, $\forall i \in \{1, \ldots ,N\}$ (since individuals do not communicate with themselves), and finally, the sum of each row is 1.
- Individual characteristics, ${\mathbf {B}} \in {\mathbb {R}}^{N \times 3}$ comprises the following three characteristics $(s_i, p_i^{Comm}, p_i^{Obs})$ for all $i \in \{1, \ldots , N\}$ agents:
  1. Suggestibility, $s_i$, is the proportion to which agent *i*, in case of receiving information, nears her beliefs to that of the sender (Accordingly, if $s_i=0$, agent *i* does not modify her beliefs at all, even when receiving information, while in case $s_i=1$, agent *i* changes her beliefs in a way to match the received data).
  2. Communication activity $p_i^{Comm}$, is the probability (or “willingness”) of agent *i* to communicate in any round (see later). Accordingly, the real, “materialising” communication between agents $i \rightarrow j$ is $c_{ij}=p_i^{Comm} \times a_{ij}$.
  3. Observation activity, $p_i^{Obs}$, is the probability (or “willingness”) to check up on data personally.

Our question is the following: what are the features of an “optimal” group (in terms of its communication network ${\mathbf {C}}=(c_{ij})$ and individual properties ${\mathbf {B}}$) in case “optimal” refers to the ability of (i) reaching high level of consensus within a certain amount of time, (ii) gaining accurate information within a certain amount of time, and (iii) creating a consensus concordant to the environment, within a certain amount of time [that is, when both (i) and (ii) are equally important].

In order to answer these questions, we have optimised the above parameters (the communication network and the individual characteristics) by a genetic algorithm (For the detailed flowchart and the parameter settings of the optimisation algorithm see the “Methods” section). The fitness function—determining what “optimal” means—is defined as

1 $$\begin{aligned} F = \alpha - \kappa , \end{aligned}$$

where $\alpha$ denotes the performance of the group, and $\kappa$ refers to the costs associated to the activities.

The performance of the group, $\alpha$, in accordance to the three definitions of being “optimal”, can refer to the

(i) achieved *accuracy* of the group, $\alpha ^{Acc}$
(ii) level of consensus that has been reached, $\alpha ^{Cons}$, and
(iii) both (i) and (ii) with equal weight: $0.5 \times \alpha ^{Acc} + 0.5 \times \alpha ^{Cons}$.

The achieved accuracy, $\alpha ^{Acc}$ refers to the *ratio* of the initial group-error, $\gamma ^{Init}$, that has been corrected during the run:

2 $$\begin{aligned} \alpha ^{Acc} = \frac{\gamma ^{Init} - \gamma ^{Final}}{\gamma ^{Init}} \end{aligned}$$

and, similarly, the group performance related to consensus, $\alpha ^{Cons}$, is the ratio with which the initial disagreement has been reduced:

3 $$\begin{aligned} \alpha ^{Cons} = \frac{\delta ^{Init} - \delta ^{Final}}{\delta ^{Init}}. \end{aligned}$$

The disagreement, $\delta$, is simply the mean standard deviation among the members’ belief vectors (taken for all the *K* elements, and then averaged). In order to keep the two values, (the error $\gamma$, and the disagreement $\delta$) comparable, the group-error has been calculated in a similar way: it is the average deviation between the belief vectors and the environment vector, taken for all the *K* elements, and then averaged:

4 $$\begin{aligned} \gamma = \langle \langle \sqrt{(BeliefVect_k^{(i)} - EnvironmentVect_k)^2} \rangle _k \rangle _i = \sum _{i=1}^{N} \sum _{k=1}^{K} \frac{\sqrt{(BeliefVect_k^{(i)} - EnvironmentVect_k)^2}}{N \times K}, \end{aligned}$$

where $<...>_k$ and $<...>_i$ denotes averaging over the $k \in \{1, \ldots ,K\}$ elements and $i \in \{1, \ldots ,N\}$ agents, respectively.

The costs, $\kappa$, in Eq. (1) is the total cost of the activities: it is the mean communication activity $\langle p_i^{Comm} \rangle _i$ multiplied by the cost of communication $\kappa ^{Comm}$, plus the mean observation activity $\langle p_i^{Obs} \rangle _i$ multiplied by the cost of observation $\kappa ^{Obs}$:

5 $$\begin{aligned} \kappa = \langle p_i^{Comm} \rangle _i \kappa ^{Comm} + \langle p_i^{Obs} \rangle _i \kappa ^{Obs}. \end{aligned}$$

Since genetic algorithms *maximize* the fitness function, Eq. (1) expresses the requirement of achieving high group performance ($\alpha$) on the lowest possible costs ($\kappa$). Here we mention that in the theoretical case when the performance of a group *decays* during a run, $\alpha$ can be negative as well (which would happen in case $\gamma ^{Final}>\gamma ^{Init}$ in Eq. (2) or $\delta ^{Final}>\delta ^{Init}$ in Eq. (3)). However, since communication decreases the level of disagreement $\delta$ and observation lowers the estimation error $\gamma$, in practice we always obtained positive $\alpha$ values. Accordingly, we can conclude that the first term of the fitness function, $\alpha$, is positive and—since being a ratio—takes values from the [0, 1] interval.

The other term, $\kappa$ (defined by Eq. (5)) takes values from the $[0, \kappa ^{Comm}+\kappa ^{Obs}]$ interval, that is, its maximal value depends on the actual set of parameters. This term, corresponding to the costs, *decreases* the final fitness value. In the theoretical case when $\kappa > \alpha$, the fitness value can be negative as well. Accordingly, the fitness function *F* takes values from the $[-(\kappa ^{Comm}+\kappa ^{Obs}), 1]$ interval.

## Results

Our results indicate that consensus and well-informedness require different group and individual properties. In the following subsections we will overview the features promoting one or the other, as well as the characteristics promoting both requirements simultaneously. For sake of lucidity, in all of the figures, we mark the data referring to well-informedness with red colour, the data corresponding to consensus with blue colour, and the data referring to the results gained by optimising on both of these aspects, with green colour. Since those group and individual properties which satisfy the requisites of both consensus and well-informedness (marked with green curves) are always in between the two characteristic features, we focus on the results referring to well-informedness and consensus.

Since studies related to the optimal size of real-life decision-making groups agree that the optimal number is between 5 and 30^25–27^—depending on the specific goals as well^2,28,29^—we have chosen the size of the groups, *N*, to be between 5 and 30. In order to test the robustness of our results, we have performed the optimization for a wide range of parameters, and found that the main conclusions hold, independently from the topical settings. (For details see the Supplementary Information). However, in order to delineate our results we had to choose a certain set of parameters, which is the following: $N=20, K=20$, Cost of communication $\kappa ^{Comm} = 0.05$, Cost of observation $\kappa ^{Obs} = 0.5$, and finally, the number of rounds in each run, *R* (determining the “time” each group has for reaching consensus and/or for gathering information) is $R=100$ (discussed in detail later).

**Figure 2:**
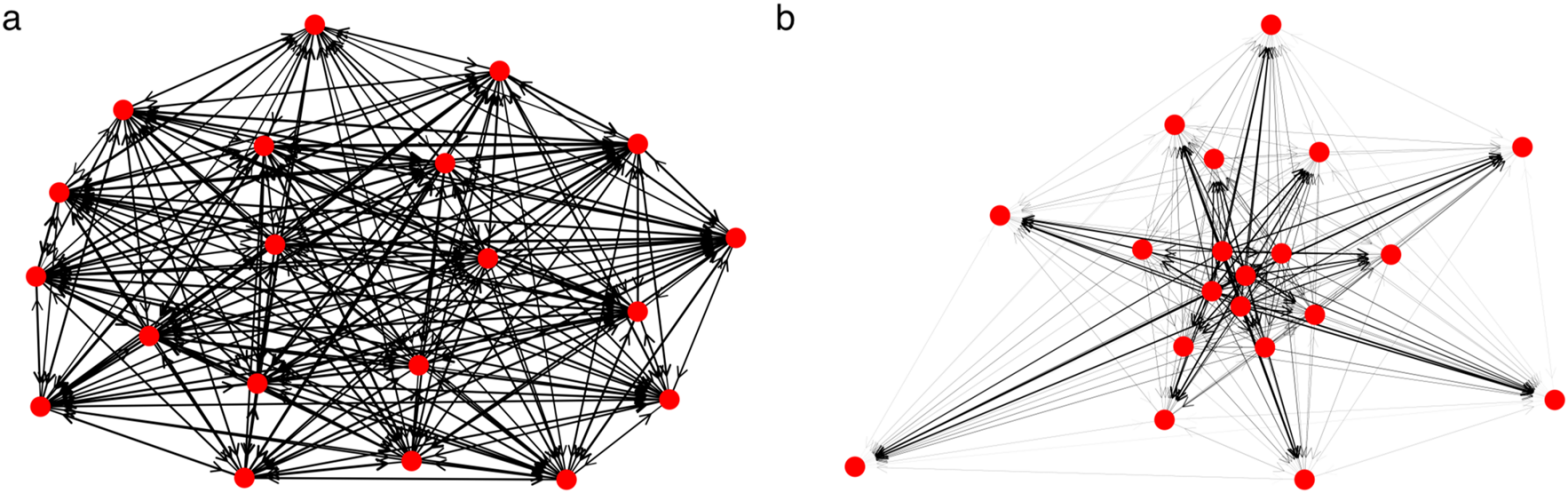
Visualization of optimal communication networks, in case “optimal” refers to a structure promoting (**a**) *consensus* or (**b**) *well-informed members* in case of high access to information (high values of *H*). The communication network depicted in sub-figure (**a**) is a full graph with hierarchy value 0. In such networks all agents participate in the circulation of information to the same extent. In contrast, the hierarchy value of a communication network promoting well-informedness is close to 1 (**b**). In this case, some agents become “influencers”: individuals transacting the bulk of the information circulation (nodes located in the center of the graph), and some become “listeners”: agents receiving information, but not participating in its circulation (nodes on the periphery).

### Comparison of the optimal network structures

The differences between the optimal network structures promoting on the one hand *consensus*, and, on the other hand *well-informed* members, are apparent: The one promoting the emergence of *consensus*, by and large, follows intuition: the most effective networks are full graphs (see Fig. 2a) in which nodes—representing individuals – participate in the circulation of information equally. In contrast, the one supporting well-informed members is highly hierarchical in which individuals differentiate according to their role in information circulation (Fig. 2b) (Hierarchy is defined as the fraction of edges not participating in cycles in a directed graph^30^).

**Figure 3:**
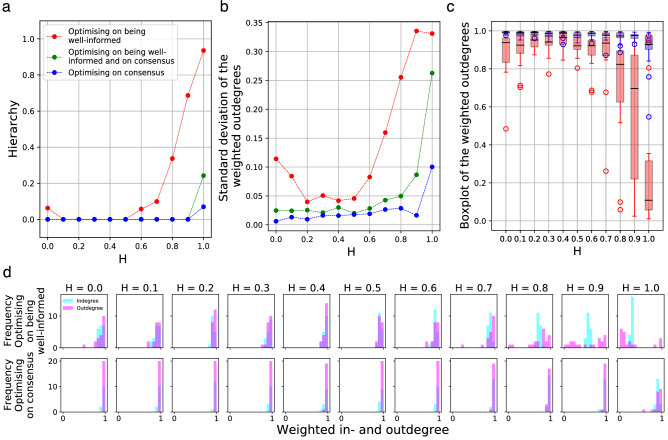
Features of the optimal communication networks, as a function of *H*, for groups optimised on consensus (blue curves), on well-informedness (red lines) and on both aspects (green markers). (**a**) Communication networks ensuring well-informed members are more hierarchical above a certain value of *H* (access to information) than communication networks promoting consensus. Interestingly, this phenomenon enhances with *H*, indicating that in case the goal is to become well-informed, *better* access to information promotes *more* hierarchical communication networks. Green curve: the hierarchy levels of the communication networks promoting both requirements resemble more to the ones facilitating consensus (For the slight increase of the blue curve at $H=1$, and of the red curve at $H=0$, see Supplementary Information). (**b**) The standard deviation of the weighted out-degrees for the three cases, as a function of *H*. Weighted out-degrees reflect the extent to which agents participate in the circulation of information. Accordingly, the higher the standard deviation, the bigger the difference among the agents regarding their participation in the information-circulation. (**c**) Visualization of the same phenomenon using boxplots of the weighted out-degrees. The horizontal bars on the boxes represent the median communication activity (see also Fig. 4a), the surrounding areas denote the interquartile ranges, and finally the dots represent the outliers [in accordance with the histograms shown on sub-figure (**d**)]. The interquartile ranges are significantly larger in case the group is optimized for being well informed (marked with red color) than for reaching consensus (marked with blue) primarily at high values of *H*. (**d**) Histograms of the weighted in-degrees (light blue) and out-degrees (magenta) for all the 11 values of *H*. Top row: in case the aim is to become well-informed, as *H* grows, agents become more and more specialised (diverse) with respect to their information circulation activities (which is basically their communication activity) but remain very homogeneous regarding the amount of received information. Bottom row: in case the aim is to reach consensus, agents are homogeneous, both regarding the amount of sent and received information.

**Figure 4:**
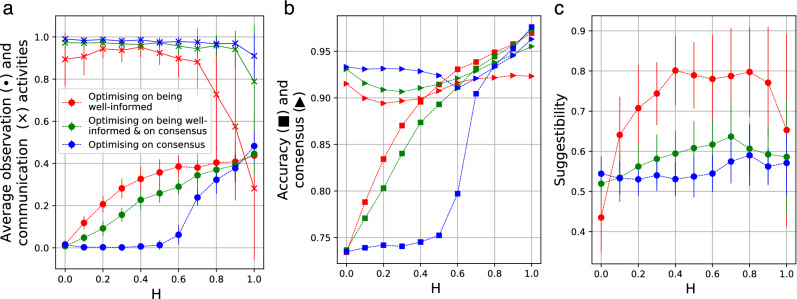
Optimal level of individual characteristics, as a function of *H*. (**a**) The average amount of observation and communication activities, according to the three definitions for “optimal”. In case the aim is to reach *consensus*, intense communication is needed (blue ’x’ signs), but at higher values of *H* (good access to information) the optimal strategy becomes to observe the external source directly. In case the goal is to become *well-informed* (red curves) the better the individuals’ access to information, the *more* they should observe. At smaller values of *H* the communication is very intense (red ’x’ signs), but this activity can not compensate the high *in*accuracy originating from the poor access to information: as it can be seen in sub-figure (**b**) the accuracy of the groups (small squares) are closely related to their average observation activities. Interestingly, at very high values of *H*, groups aiming to reach merely *consensus* can become better-informed than groups aiming to become well-informed (when consensus is reached via the observation of the external environment directly). This is due to the fact that, if the members of a group possess accurate factual information then they are necessarily in consensus as well (see the small triangles), while the opposite is not true: in case members of a group are in consensus, they do not not necessary hold accurate information (blue lines at $H<0.5$). In order to make this phenomenon more visible, we have reduced the size of the symbols (squares and triangles) in sub-figure (**b**). (**c**) Although intuition suggests that consensus requires suggestible people, while aiming for accurate information requires more self-willed individuals, our results suggest the opposite: individuals do not need to be suggestible in order to reach consensus (blue curves), but to become well-informed (red curves). In all three sub-figures the green lines—marking the optimal values for groups aiming to be both accurate and well-informed—are always in between the blue and red lines, indicating that such groups simply try to balance between the two requirements by choosing “in-between” values. The vertical lines on sub-figures (**a**) and (**c**) refer to the standard deviation among the group members.

This characteristics can be seen from the distribution of the weighted in-degrees and out-degrees: the weighted *out*-degree of a node reflects the given node’s activity in *sending* information, that is, in its circulation, while the weighted *in*-degree indicates the amount of *received* information. For example if the weighted out-degree of a node is close to 1, while its weighted in-degree is small (close to 0), then this node sends lot of information but does not receive any (The weighted out-degree of a node is basically its communication activity). As it can be seen in the bottom row of Fig. 3d, both the weighted in-degrees (marked by light blue color) and out-degrees (marked by purple) are $\approx 1$ basically for all nodes, marked by a sharp peak around 1, independently of the value of *H*. In other words, independently of the ratio of the information that is accessible for the individual agents, in case the goal is to reach consensus, the optimal communication network is a full graph with nodes (agents) participating equally in the information circulation, regarding both “talking” and “listening”.

At first sight, the independence of *H* seems to be reasonable since in case the aim of the group is merely to reach consensus (without the requisite of achieving accurate information) *H* does not seem to be important, since observation itself does not seem to be important. However, as we will see in the next subsection, in case of higher *H* values ($H > \approx 0.6$, slightly depending on the parameters), even in cases when accuracy does not matter, it is worthy to observe the environment directly instead of merely communicating, despite the fact that observation is a many times more costly activity than communication. This phenomenon enhances with the growth of *H*.

According to our simulations, achieving accurate information requires a much more structured group, referring to a more hierarchical communication network (see Fig. 3a, red curve) and more specialised individuals (Fig. 3d, top row). Interestingly—at least contrary to intuition—the increase of hierarchy and specialisation *grows* with *H*, that is, *better* access to information promotes *more* hierarchical communication networks and more specialised individuals.

Independently of the topical parameter settings, the following observations can be made: for *small* *H* values ($0<H<0.6$), the structure of the optimal communication network—but not the agents’ properties—is similar to the one promoting consensus: it is a full graph, like the one in Fig. 2a. However, around $H >\approx 0.5$ (slightly depending on the parameters), its hierarchy level starts to increase sharply, and around $H=1$ it takes a value close to 1 (Fig. 2b).

This increase of hierarchy is due to the *specialisation of agents with respect to their participation in the circulation of information*: as it can be seen in Fig. 3b (red curve, showing the standard deviation of the weighted out-degrees), Fig. 3c (depicting the boxplots of the weighted out-degrees) and Fig. 3d (top row, depicting the histograms of the weighted in-degrees and out-degrees), from around $H >\approx 0.6$ more and more agents decrease their activity in the circulation of information. At $H >\approx 0.8$, some individuals entirely cease to initiate communication (marked by the growing amount of magenta-coloured bars around zero). Finally, at $H=1$, *most* of the agents are silent, but still receiving information, which can be known from the histogram of the in-degrees (marked with light blue colour in the top row of Fig. 3d), showing that there are no agents with in-degree values close to zero. In these cases, the flow of information is ensured by a small minority, whose weighted out-degree values (marked with magenta colour) are still close to 1.

These observations are valid independently from the parameter settings, except for one case: if the cost of communication is higher than $40 \%$ of the cost of observation ($\kappa ^{Comm} \ge 0.4 \times \kappa ^{Obs}$), then the communication network falls apart, because in such cases it is not worth anymore to maintain communication (for more details see the Supplementary Information), and accordingly, agents will not be connected anymore.

**Figure 5:**
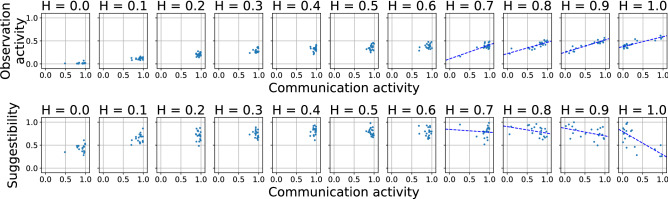
Correlation of the individuals’ features. Top row shows how the observation activity correlates to the communication activity, and the bottom row depicts how the suggestibility is related to the communication activity. Each dot represents an individual in a group optimised on becoming well-informed. Above a certain value of *H* individuals start to specialise on different roles: in an optimised group, individuals with higher communication activities tend to be more active regarding their observation activity as well, but, at the same time, tend to be less suggestible.

### Optimal individual characteristics

#### The influencers

As we have seen in the previous subsection, in case the aim is to gain accurate information, at high access to information ($H > 0.5$), with the increase of *H*, a decreasing portion of the group ensures the flow of information. We have also seen that the communication activity of these agents (which is the same as the sum of the node’s weighted out-degree) is around 1. But what can be known about the other characteristics of this minority: their observation activity and suggestibility? As it turns out, their observation activity also tends to be higher than their mates’ (see Fig. 5a), while they have a clear tendency for being less suggestible (Fig. 5b). In other words, those active agents who transact the bulk of the information circulation (the central, “high-ranking” nodes in the communication network), tend to be more active “in general”, both regarding communication and observation. At the same time, they are less suggestible. However, these tendencies appear only at higher values of *H*, since for smaller *H* values, individuals simply do not differentiate. In short: in case members of a group aim to gather accurate information regarding an external environment, if individuals have *high* access to information, in an optimal group “influencers” appear who are more active both in circulating the information and in making observations, but, at the same time, are less suggestible.

#### The optimal amount of activities in case the aim is to reach consensus

If a group is to reach *consensus*, its members have to *communicate* intensively. This relation shows up in Fig. 4a as well, in which the blue ’x’ signs (referring to the average communication activity $\langle p_i^{Comm} \rangle _i$ within an optimised group) are close to 1 for all values of *H*. $p_i^{Comm}=1$ means that the probability of communication initiated by agent *i* in any round is 1. It is only at $H=1$ (full access to information) when this value drops from 1 to $\approx 0.9$, which decrease—most probably—results from the *intense increase* of the optimal *observation* activity (marked by filled blue circles in Fig. 4a). According to this curve, in case individuals have access to a large ratio of the data (high values of *H*), then the optimal strategy to reach *consensus* (without the requisition of holding precise information!) is to observe the “external world” directly, despite the fact that observation is much more costly than communication. This situation, for example, can refer to cases when consensus emerges more easily by accessing directly a publicly and fully available source, then merely relying on communication. This strategy remains the optimal one as long as the circumstances are such as that agents are able to gain accurate information by accessing the external sources. Such a circumstance—apart from too small *H*—can be violated due to *too short time compared to the complexity of the external source* as well (that is, if *R* is small compared to *K*, see Supplemetary Fig. 3.7). In other words, if the external source is too complex (large database, long book, etc.) compared to the time given (say, a few hours) then the best option remains to be the intense communication; otherwise the optimal strategy is to refer all group members to the external source, again, even in cases accuracy does not matter and observation is costly.

#### The optimal amount of activities in case the aim is to become well-informed

According to our results, the better the individuals’ access to information, the *more* they should observe: as it can be seen in Fig. 4a, the optimal amount of average observation (filled red dots) *increases* with the growth of *H*. This phenomenon originates from the fact that even “full communication”—marked with red ’x’ symbols, which are between 0.9 and 1 for all *H* values smaller than 0.8—can not compensate for the small access to information. This inability of becoming well-informed shows up very clearly in Fig. 4b which depicts the accuracy (or “well-informedness”) of the group (small red squares); the strong correlation between the observation activities (filled dots in Fig. 4a) and the accuracy of the groups (small squares in Fig. 4b) is apparent. On the other hand, with the increment of observation, the optimal amount of communication decreases, (marked by the decline of the red curve with ’x’ symbols in Fig. 4a) referring to the shift of the optimal strategy from intense information-sharing (by communication) to a strategy in which personal information gathering is accompanied by the appearance of “influencers” (individuals transacting the bulk of the information circulation).

#### The role of suggestibility

Interestingly enough, individuals do *not* need to be suggestible in order to reach consensus; suggestibility is needed to become well-informed. Figure 4c shows the optimal level of the average suggestibility values as a function of *H*, for the following tree cases: when the aim of the group is (i) to reach consensus (blue line), (ii) to have well-informed members (red curve), and (iii) when both of these aspects are equally important (green line). Although intuition suggests that consensus requires suggestible people, while aiming for accurate information requires more self-willed individuals, our simulations suggest the opposite: the blue line is between approximately 0.5 and 0.6 for all values of *H* indicating that a moderate amount of suggestibility serves the emergence of consensus the best. In other words, consensus emerges fast when people weight approximately equally between their private and social information.

In contrast, if the aim of the group is to have well-informed members, agents have to be considerably more suggestible (except when $H=0$, that is, when agents do not have access to information at all). Furthermore, this dependency is not monotonic, since at high values of *H* ($H > 0.8$) the optimal amount of average suggestibility starts to decrease again. We assume that this phenomenon is related to the high accuracy that can be achieved (Fig. 4b, small red squares) without intense communication (Fig. 4a, red ’x’ symbols). In other words, in these cases, a group does better if its members look after the data themselves and stick to their own observations.

## Discussion

Studying the circumstances by which consensus—or fragmented opinion clusters^31–33^—emerge has created an entire scientific field known as “opinion dynamics”^14,34,35^. In the related models, agents usually form their beliefs (opinions) based exclusively on communication with their peers. In contrast, in real-life systems, individuals are usually embedded into an external environment which they can observe, at least partly, and the content of communication—particularly in case of decision making—is usually related to data referring to this external world^36^.

In the present study we incorporate these real-life features by creating a model in which agents are embedded into an external environment which they can observe (partly, controlled by a parameter *H*) and share their information by communication. Our aim is to answer the following question: what are the characteristics of the *optimal* groups, if by “optimal” we mean that group-members can (i) reach a high level of consensus, (ii) become well-informed with respect to the external “world”, and (iii) satisfy both of these requirements with equal importance. Each group is characterised by its communication network and the features of its members: their communication activity $p_i^{Comm}$, observation activity $p_i^{Obs}$ and suggestibility $s_i$.

We find that the optimal group structures differ from each other in several ways and that “optimal group properties” (which are discussed in detail in the manuscript) can be associated to the various requirements. These results can facilitate the optimal composition and functioning of groups under the pressure to make effective decisions, and can serve as a guideline in organizing groups aiming to reach consensus or gather accurate information. Furthermore, these findings—especially the ones related to suggestibility—can shed new light on psychological phenomena as well. For example, another reason why little children are very suggestible^37^ might be related to the extreme speed with which they have to learn at this age.

**Figure 6:**
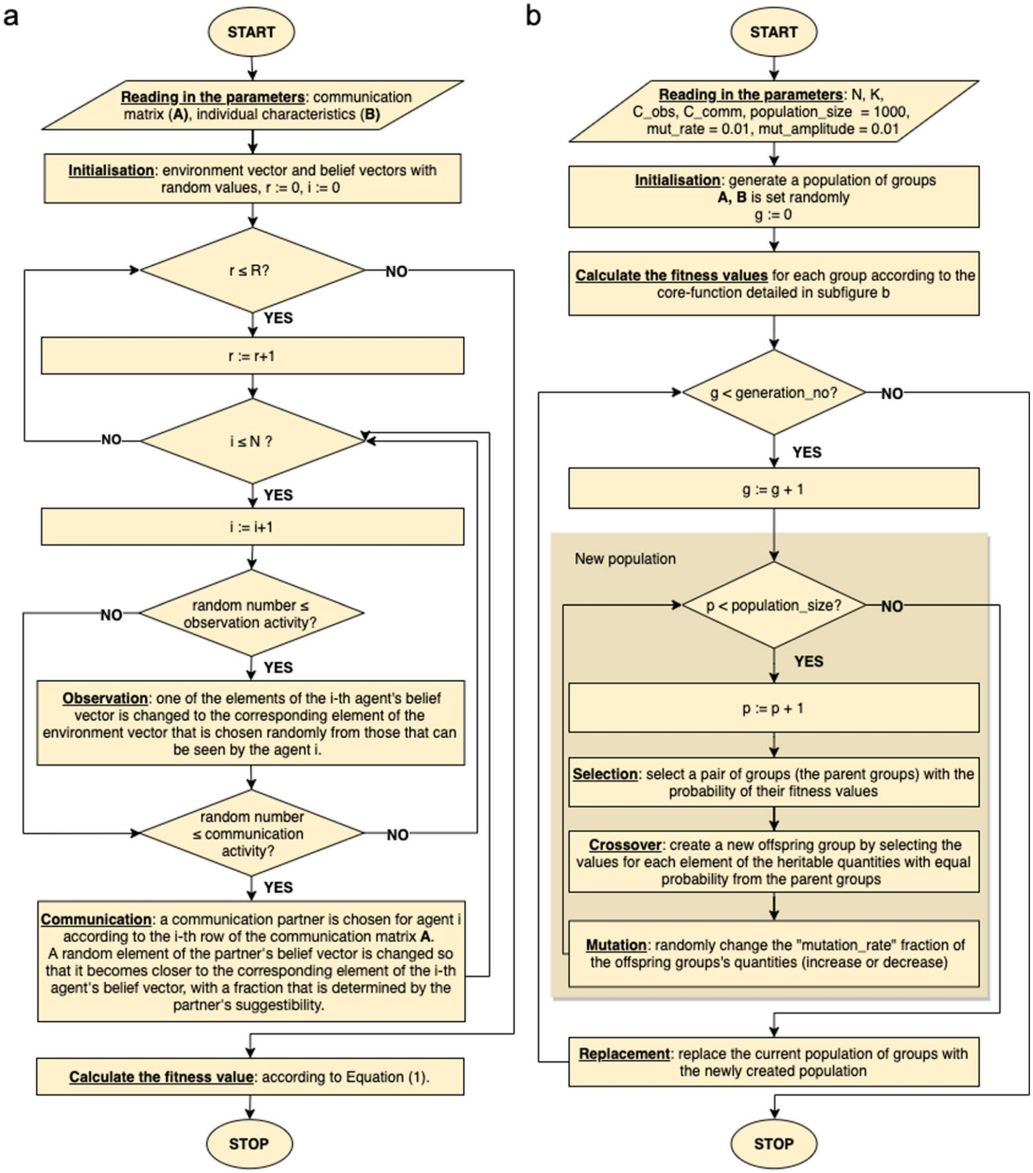
The flowchart of the algorithm. (**a**) The flowchart of the core-function, referred to as “Run”. Each run consists of *R* rounds, during which all $i \in \{1, .., N\}$ agents communicate and/or make observations with probabilities $p_i^{Comm}$ and $p_i^{Obs}$ respectively. At the end of the method, a fitness value is being calculated, reflecting the quality of the input parameters. (**b**) The *genetic algorithm* uses these fitness values to generate the newer and newer “generations” which are assumed to be more and more optimal. “Optimality” is defined by the fitness function (see Eq. (1)).

## Methods

The code was written in Python. It consists of two main parts:

i A “core-function” (referred to as “Run”) consisting of several rounds during which a group of individuals aims to reach consensus, gather accurate information or aims to reach both of these goals (depending on the fitness function). Figure 6a depicts the flowchart of this method.
  The *input* of this function are the parameters that we want to optimise: the communication network in the form of an adjacency matrix ${\mathbf {A}}=(a_{ij})\in {\mathbb {R}}^{N \times N}$ and the characteristics of the individuals, ${\mathbf {B}} \in {\mathbb {R}}^{N \times 3}$ (comprising the suggestibility value $s_i$, communication activity $p_i^{Comm}$, and observation activity $p_i^{Obs}$ for all $i \in \{1, \ldots , N\}$ agents).
  The *output* of this function is the *fitness* value defined by Eq. (1), indicating how optimal the input parameters are.
  Each run consists of *R* rounds. Accordingly, *R* defines the “time” the group has in order to reach its goal (consensus, well-informedness or both). At the beginning if each round (in the Initialisation step) the environment vector, along with the belief vectors, are set randomly with values taken from the [0, 1] interval with uniform distribution. In each run, after the initialisation, in all of the *R* rounds, each agent *i* communicates and/or makes observations with the probability defined by their $p_i^{Comm}$ and $p_i^{Obs}$ values, respectively. In case of communication between individuals $i \rightarrow j$, a randomly chosen element of agent *j*’s belief-vector assimilates to that of agent *i*’s, with the extent (ratio) of agent *j*’s suggestibility value $s_j$. In case agent *i* observes a certain element of the environment vector (to which (s)he has to have access to), the corresponding element of her belief vector is changed to the “accurate” value.
ii The optimisation has been carried out by a genetic algorithm^38^ whose flowchart is depicted in Fig. 6b. The inputs of this function are, from the one hand, a particular set of the parameters characterising the run (such as *N*, *H*, *K*, *R* and the costs), and, on the other hand, the parameters tuning the genetic algorithm itself: the so called “population size”, defining the number of “solutions” (or “chromosomes”) in each generation, the ratio of mutations mut_rate, the amplitude of mutations mut_amplitude, and the number of generations gen_no.
  A “chromosome” is basically a set of parameters whose type and size agree with the one we want to optimise (in our case ${\mathbf {A}}$ and ${\mathbf {B}}$, a set of features defining a group) but is not necessary optimal. In practice, usually, at the beginning of the optimisation, the population_size occurrence of chromosomes are set randomly. The optimal value of the parameter population_size is defined by the balance of two aspects: on the one hand, higher values for population size ensures more divers solution candidates in each generation, and, accordingly, renders the appearance of more optimal results probable, but, on the other hand, slows down the process of optimisation as well^39,40^. Keeping this in mind, we have set this parameter population_size = 1000. The parameter mut_rate sets the ratio of the modified (perturbed) parameters after the crossover, which are perturbed with a value no bigger than mut_amplitude. And finally, the parameter gen_no defines the number of generations during which the saturation of the fitness function is ensured, that is, the rounds of optimization during which an optimal set of parameters is found. We have set these parameters as mut_amplitude = 0.01, mut_rate = 0.01 and gen_no = 900.
  Once the optimization algorithm has terminated, the *features of the optimal group* was defined as next: the last generation—as each generation—comprised population_size = 1000 chromosomes. These chromosomes (each describing a group in the form of a communication matrix ${\mathbf {A}}$ and individual features ${\mathbf {B}}$) were very similar to each other *due to the crossover of the communication matrices* (In case of the optimisation of independent parameters, the entities in the last generation are not necessarily similar). Due to this similarity it was well-reasoned to average the chromosomes of the last generation in order to yield *the* optimal parameters (In order to justify the averaging, we have checked the similarity of the chromosomes within the last generations with other methods as well).

In order to perform the optimisations, we have used a high-performance supercomputer on which optimizations can be run in parallel. On this device, each thread (optimisation) took around two and a half days, which is approximately the time interval allowed for a job. The results delineated in the present paper (along with the material covered in the Supplementary Information) is the summary of optimizations in the order of a hundred.

## Supplementary information

Supplementary Information.

## Data Availability

*Accession codes* All data generated and analysed in the manuscript are reproducible based on the algorithms detailed in the article (see the “The model” and the “Methods” sections).
