# Supplementary material for: Optimal collective decision making: consensus, accuracy and the effects of limited access to information
Source: Sci Rep. 2020 Oct 12;10:16997. doi: 10.1038/s41598-020-73853-z (PMC7550594; doi:10.1038/s41598-020-73853-z)
Supplement: Supplementary file 1 — Supplementary Information. [file 41598_2020_73853_MOESM1_ESM.pdf]

Supplementary Information

Optimal collective decision making: consensus, accuracy and the effects of limited access to information

Evelin Berekméri[1,2] and Anna Zafeiris[1,3,*]
[1]Department of Biological Physics, Eötvös Loránd University, Budapest, 1117, Hungary
[2]MTA-ELTE "Lendület" Collective Behaviour Research Group, Hungarian Academy of Sciences, Eötvös Loránd University, Budapest, 1117, Hungary
[3]MTA-ELTE Statistical and Biological Physics Research Group, Hungarian Academy of Sciences, Budapest, 1117, Hungary
*lanna@hal.elte.hu

The robustness of our model has been investigated within different parameter intervals. More precisely, we analysed the results of optimisation with different environment and belief vector lengths (K), group sizes (N), activity costs (both observation and communication) and with different step sizes of the core function (R). We chose the specific parameter values so that they would stay relevant for the model and fit in our computational time limit. The next four units include results with the original parameters - K=20, N=20, R=100, cost of communication = 0.5, cost of observation = 0.05 - unless it is noted differently.

In the following, the results (the group and individual characteristics) are discussed in three cases: when the aim of optimisation was performed in order to find optimal groups that prefer (i) to reach consensus (blue markers), (ii) to be well-informed (red markers) and (iii) when both consensus and being well-informed were equally important (green markers). (The latter case is typically not analysed separately since its results are between the results of the previous two cases.) In order to define how hierarchical is the communication network, we cut edges whose weights are under a certain threshold - we used a threshold = 1/N, unless stated otherwise.

1. <u>The size of the environment vector and the size of the belief vectors</u>

Parameter K defines the number of elements of the environment vector and the belief vectors - which controls the complexity of the external environment. We have run simulations with $K \in \{10, 20, 30\}$ values. The figures below show that parameter K has no significant effect on the optimal network structure and on the optimal individual characteristics.

Figure 1.1 shows the histogram of the weighted in- and outdegrees of the communication networks for every H value. Vertical labels mark the value of parameter K and whether the group optimises on being well-informed, on consensus or on both of the previous two cases equally. For groups optimising on consensus, we can observe that high in- and outdegree values are dominant independently of the value of H and K (Figure 1.1, 3rd, 6th and 9th row), suggesting that the optimal communication networks that promote consensus are full graph, agents taking part in the

intense circulation of the information to the same extent. This is also confirmed on Figure 1.2 (blue) for all K values, by the low standard deviation of the weighted outdegrees for all of the K values. However, in some special cases, such as $K < 30$ (and as we will see later, $R > 50$, $20<N<25$), minor increase in the hierarchy (Figure 1.3) and in the standard deviation of the weighted outdegrees is present for large H values ($H \geq 0.9$). This can be connected to the lightly diminishing communication activities at large H values (Figure 1.4, blue) and to the higher observation activities at higher H values ($H \geq 0.5$): when all of the agents have high access to the information, it is worth observing more intensively, even to the expense of the communication with a smaller cost. Although for more complex environments, such as in the case of $K = 30$ this effect is milder.

Groups that optimise on being well-informed have more structured, hierarchical communication networks for larger H values ($H \geq 0.5$), regardless of the K parameter (shown on Figure 1.3 by red markers) and have specialised individuals with respect to their participation in the circulation of information (Figure 1.1 1st, 4th and 7th row) for all K values. Slightly more hierarchical structure appears when there is no access to the information ($H=0$), along with smaller communication activities. This effect lessens with the growth of K. Otherwise, for other H values ($0 < H < 0.5$) the communication network for groups that optimise on being well-informed is full graph too.

Regarding the characteristics of individuals, the tendency of the characteristics curve is similar for all K values, while the exact values may slightly depend on the value of K. The optimal amount of average observation increases with the growth of H for all K (Figure 1.4), not only for groups aiming to be well-informed, but also for groups that want to achieve consensus (the former being more intense). However, the more complex the environment is (the higher K is), the more observation is needed in total for the optimal network, in both cases. Similarly, the tendency of the communication activities is alike for all K values, while with the growth of the environment complexity, the communication activities are slightly higher too, for all levels of access to information. In groups that optimise on being well-informed, agents communicate less on average.

Even despite intense communication in general, small access to information cannot be compensated, even for less complex environments (Figure 1.5), not until the observation activities increase. However, there is a clear connection between the level of accuracy the groups can achieve with these parameter settings (e.g. within this amount of time, controlled by parameter R) and the complexity of environment. Similarly, the level of consensus the groups can reach correlates with the K parameter.

Suggestibility values are higher for groups aiming to be well-informed, independently of K, and lower for consensus aiming groups, for $H > 0$ (Figure 1.6). However, at $H = 0$ only, when agents cannot observe the environment at all, their suggestibility level is lower for groups which optimise on being well-informed. With the increase of K, there is a slight decrease in suggestibility levels.

On Figure 1.7 we can see that in the groups that optimise on being-well informed, those agents who specialised on spreading the information (who have higher communication activity) at high H values, have also higher observation activity than the other members of the group, and are less suggestible (Figure 1.8), both phenomenon being independent of K.

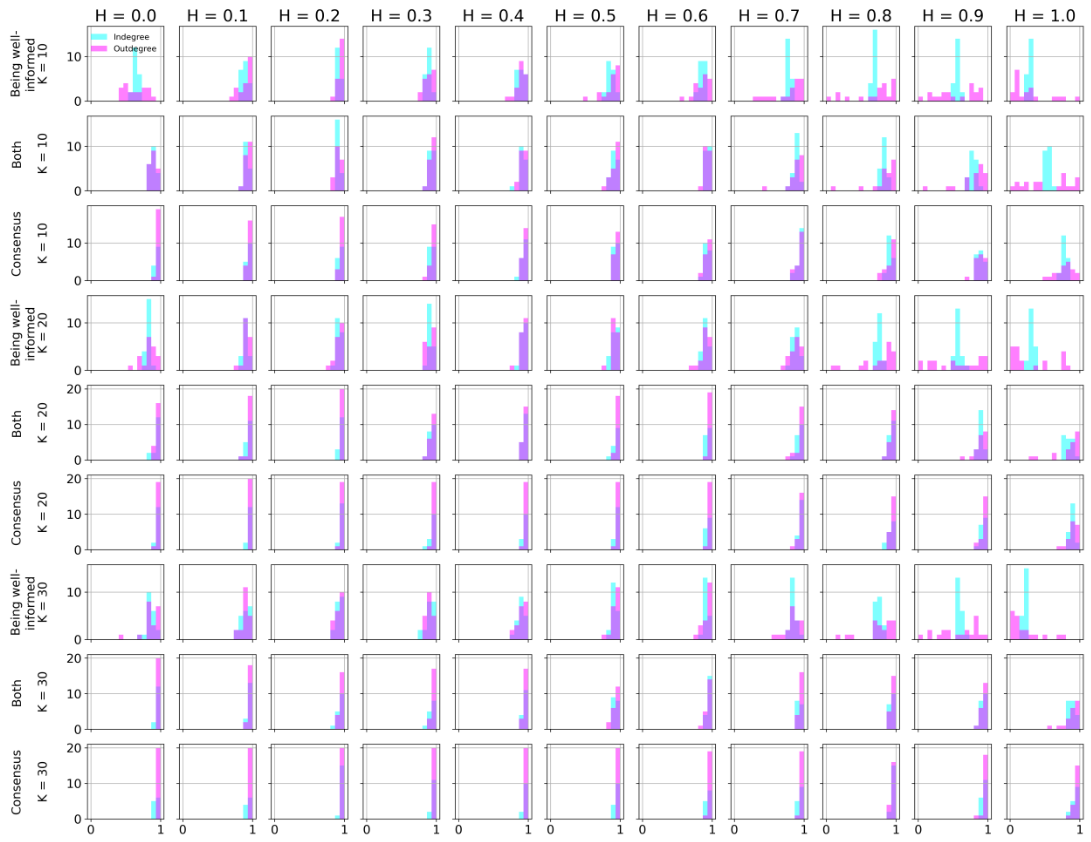

Figure 1.1. Histograms of the weighted in- and outdegrees for $H \in \{0.0,\ 0.1,\ \ldots,\ 1.0\}$ and $K \in \{10, 20, 30\}$ parameters

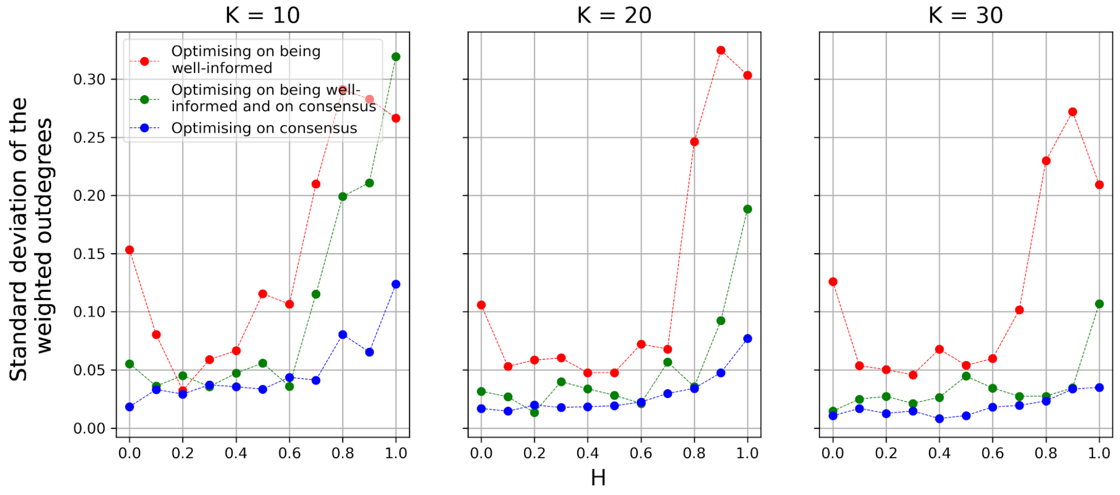

Figure 1.2. Standard deviation of the weighted outdegrees as a function of H, for $K \in \{10, 20, 30\}$

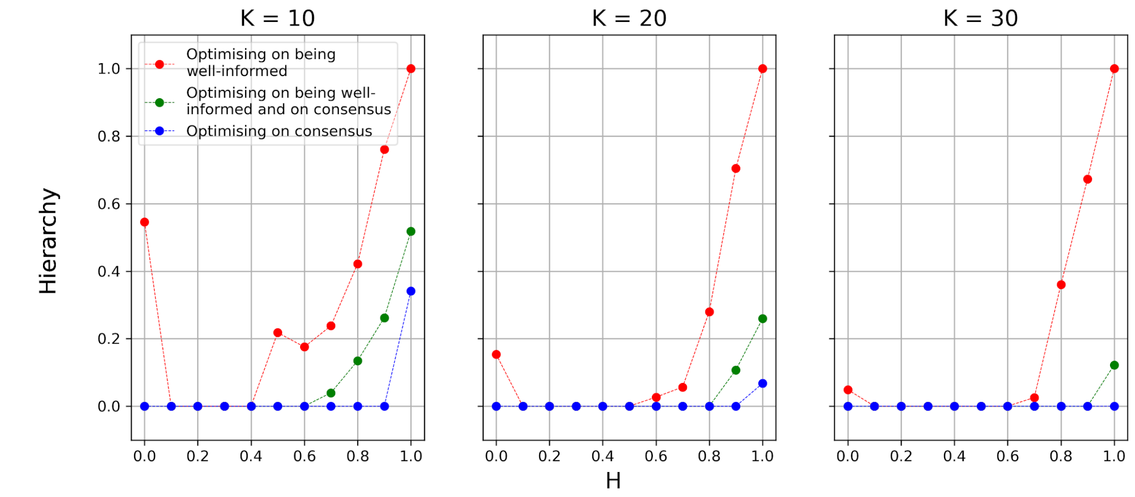

Figure 1.3. Hierarchy of the communication networks as a function of H, for $K \in \{10, 20, 30\}$

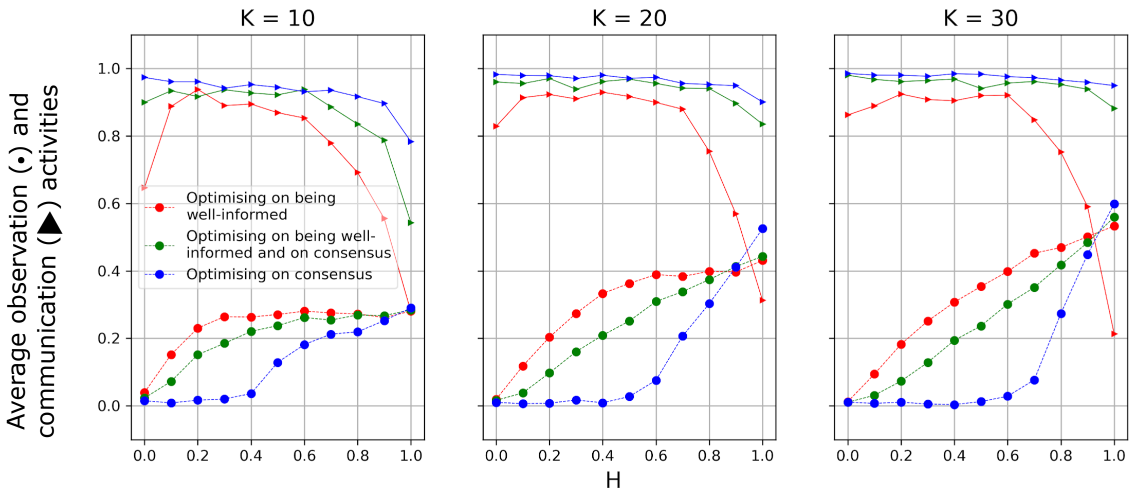

Figure 1.4. Average observation and average communication activities as a function of H, for $K \in \{10, 20, 30\}$parameters

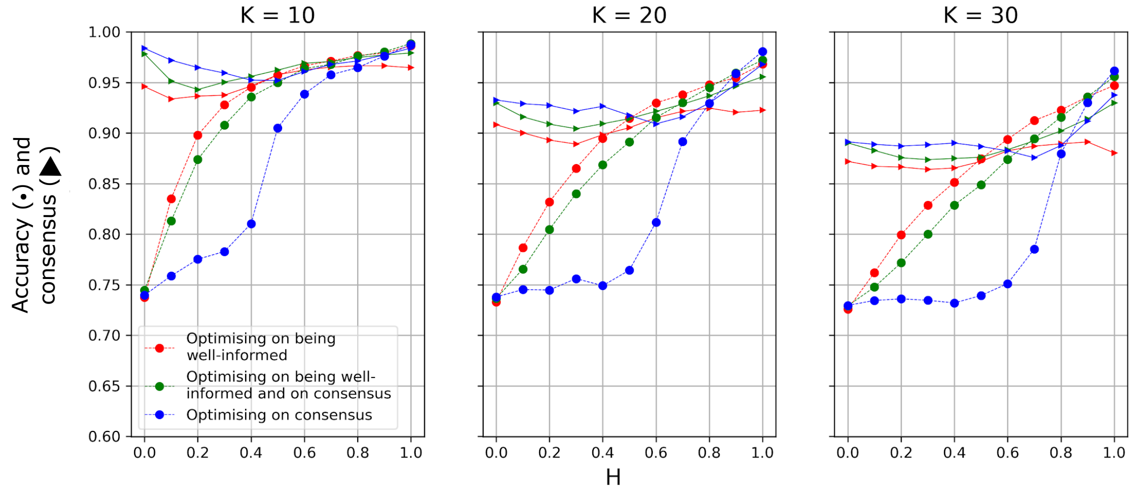

Figure 1.5. The level of accuracy and consensus as a function of H, for $K \in \{10, 20, 30\}$

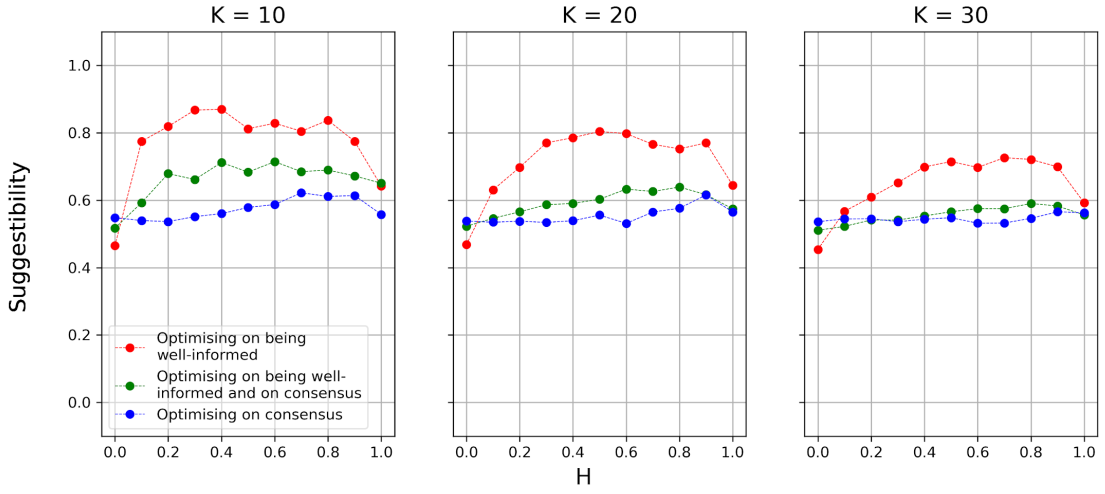

Figure 1.6. The average level of suggestibility of agents as a function of H, for $K \in \{10, 20, 30\}$

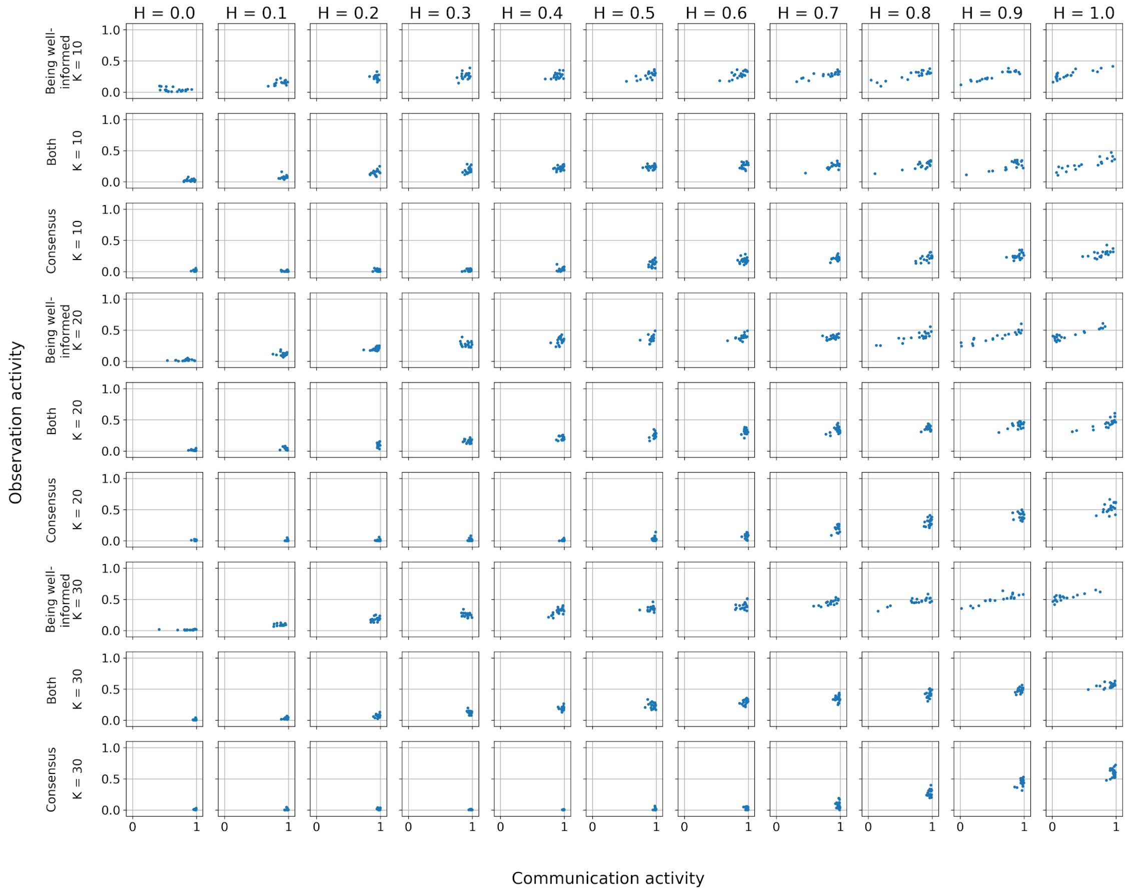

Figure 1.7. Average observation activities as a function of average communication activities for $H \in \{0.0,\ 0.1,\ \ldots,\ 1.0\}$ and $K \in \{10, 20, 30\}$ parameters

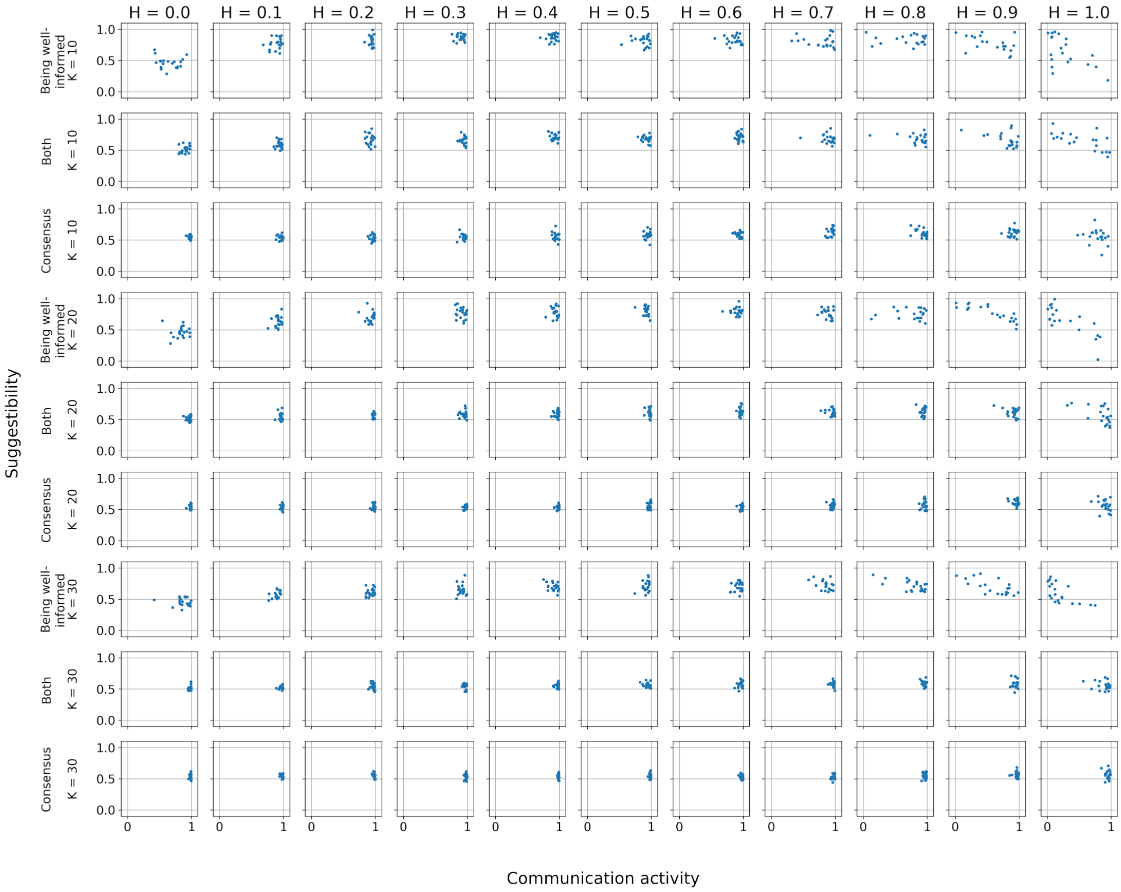

Figure 1.8. Suggestibility as a function of average communication activities for $H \in \{0.0, 0.1, \ldots, 1.0\}$and $K \in \{10, 20, 30\}$parameters

2. Group size

Parameter N marks the number of agents in a group. We have run simulations with $N \in \{5, 10, 20, 25, 30\}$ values. We used the following threshold values to calculate the hierarchy of the communication networks cca. 1/N (more precisely: 0.15, 0.09, 0.05, 0.033, 0.029, for $N \in \{5, 10, 20, 25, 30\}$, respectively). According to the figures below, changing N does not change our main conclusions.

Figure 2.1 shows that the presence of full graph, for groups aiming to reach consensus does not depend on N, nor that the agents in groups aiming to be well-informed are specialised to spreading and to receiving the information, for H>0.5. Figure 2.2 confirms this with the low standard deviation of the out-degrees in the first case and high standard deviation in the second case. Agents specialising on information spreading and on information receiving make the communication networks hierarchical, independently of N, shows Figure 2.3. Communication and observation activities behave similarly for different N values, regardless of the size of the group (Figure 2.4) and this defines the similar consensus and accuracy curves for different N parameters too (Figure

2.5). Suggestibility remains higher for groups optimising on being well-informed for all N, for H>0 (for small groups, such as N = 5, for H>0.4) - Figure 2.6. As the size of the group grows, the average amount of suggestibility decreases. Agents who specialised on spreading the information are also more actively observing the environment and at the same time they are less suggestible (Figure 2.7, Figure 2.8), regardless of the number of agents in a group.

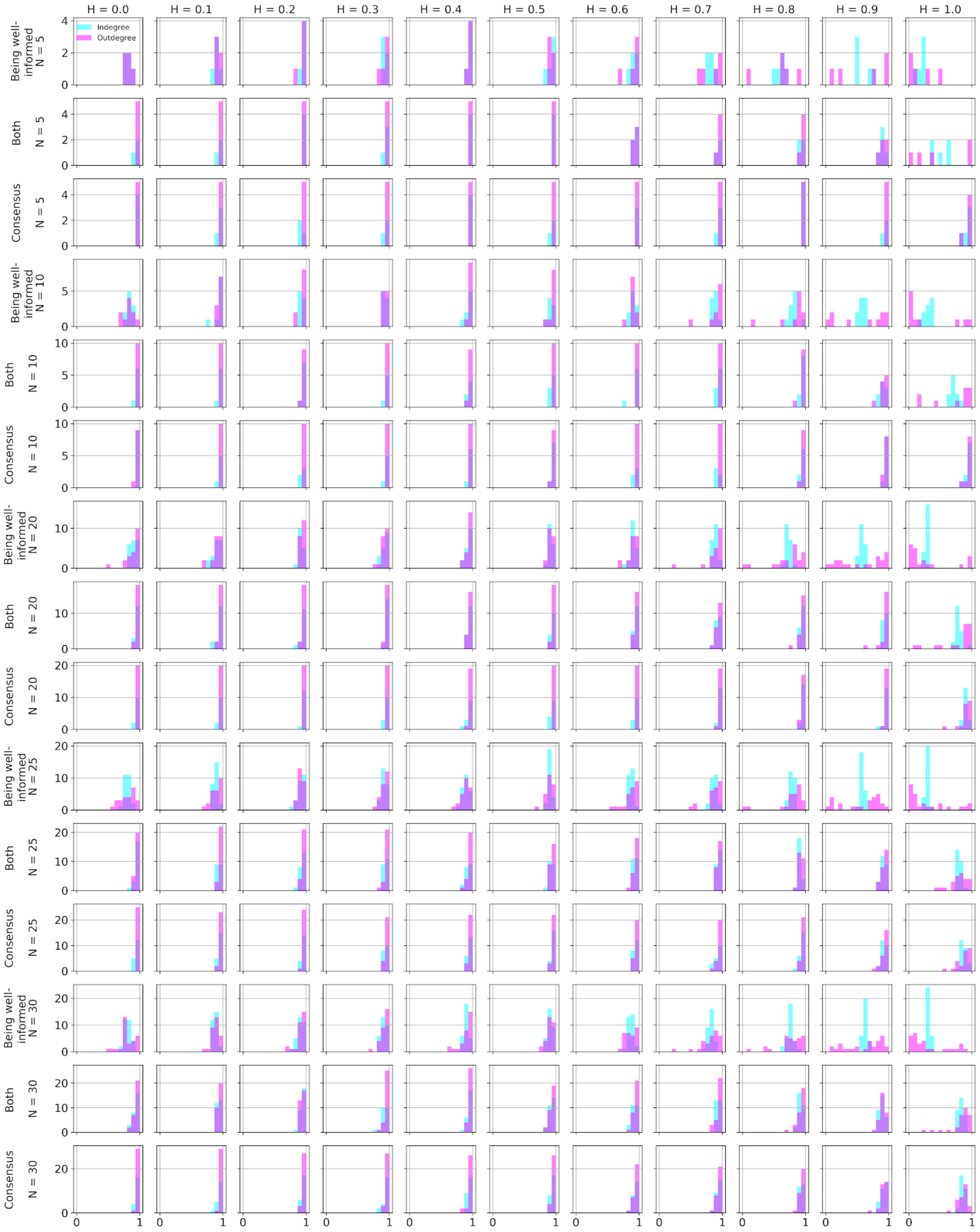

Figure 2.1. Histogram of the weighted in- and outdegrees for $H \in \{0.0, 0.1, \ldots, 1.0\}$ and $N \in \{5, 10, 20, 25, 30\}$ parameters

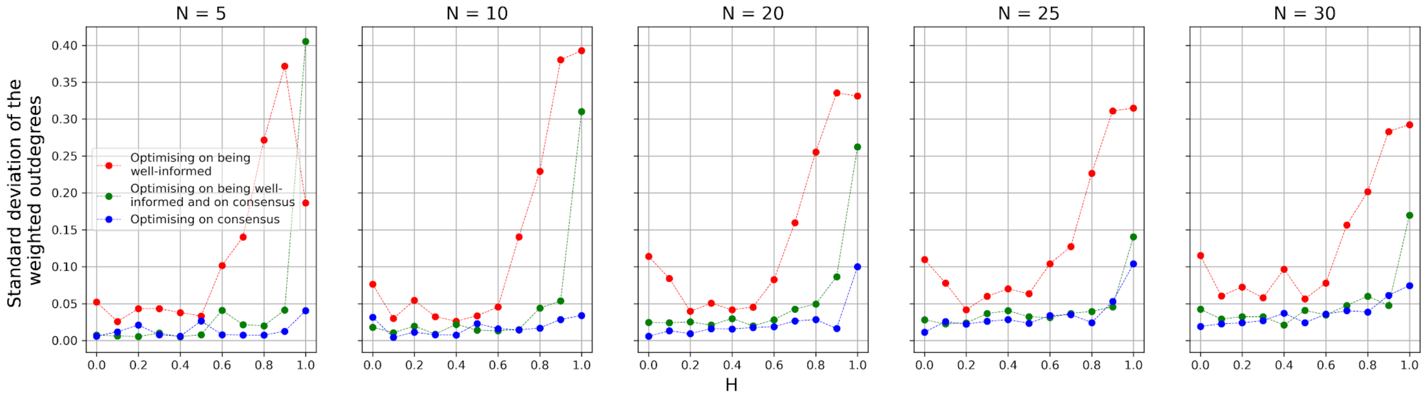

Figure 2.2. Standard deviation of the weighted outdegrees of the communication networks, as a function of H, for $N \in \{5, 10, 20, 25, 30\}$ parameters

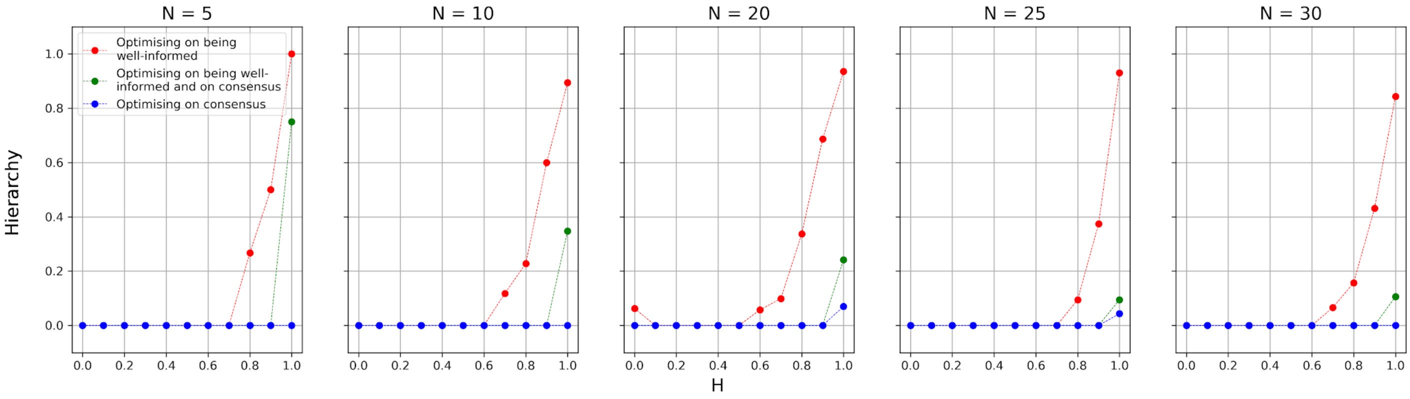

Figure 2.3. Hierarchy of communication networks, as a function of H, for $N \in \{5, 10, 20, 25, 30\}$ parameters

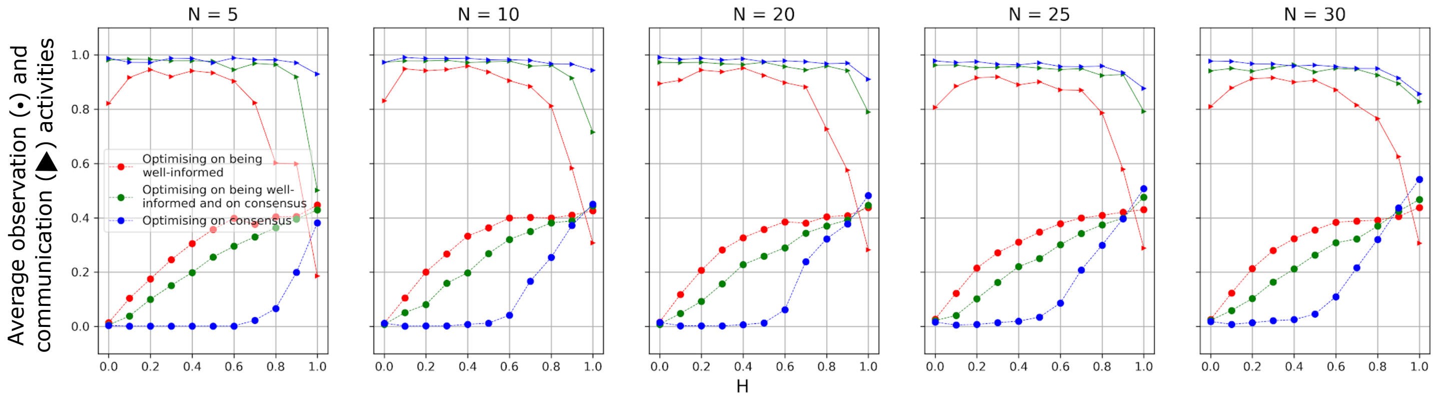

Figure 2.4. Average observation and communication activities as a function of H, for $N \in \{5, 10, 20, 25, 30\}$ parameters

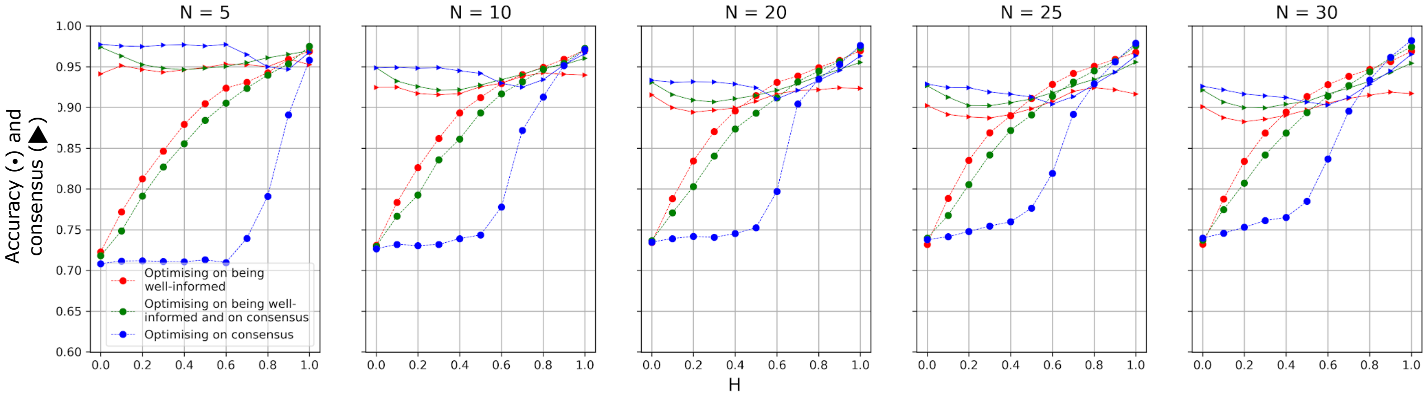

Figure 2.5. The level of accuracy and consensus the groups can reach, as a function of H, for $N \in \{5, 10, 20, 25, 30\}$ parameters

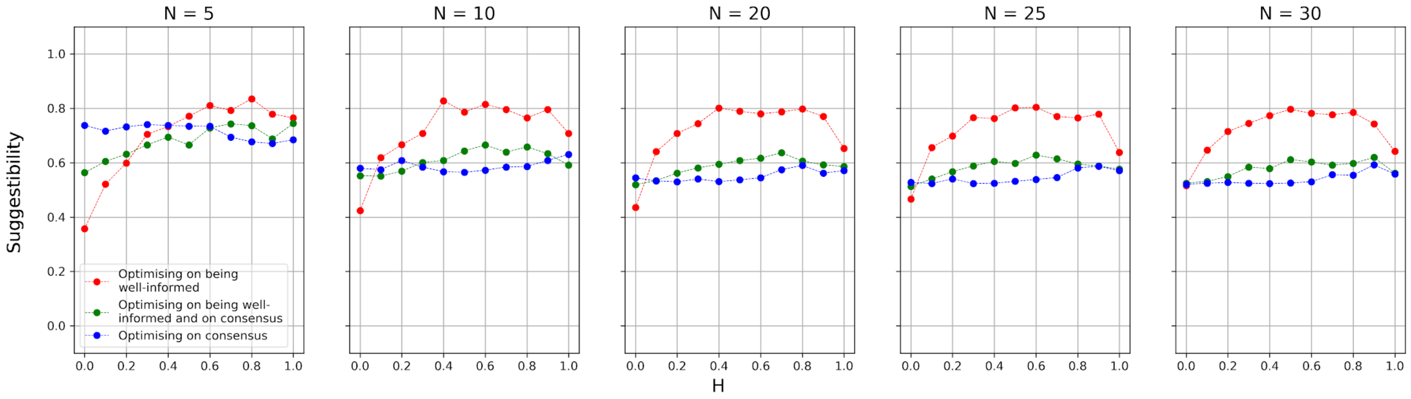

Figure 2.6. Average suggestibility levels as a function of H, for $N \in \{5, 10, 20, 25, 30\}$. In case the goal is to reach consensus (blue), as the size of the group grows the average amount of suggestibility decreases.

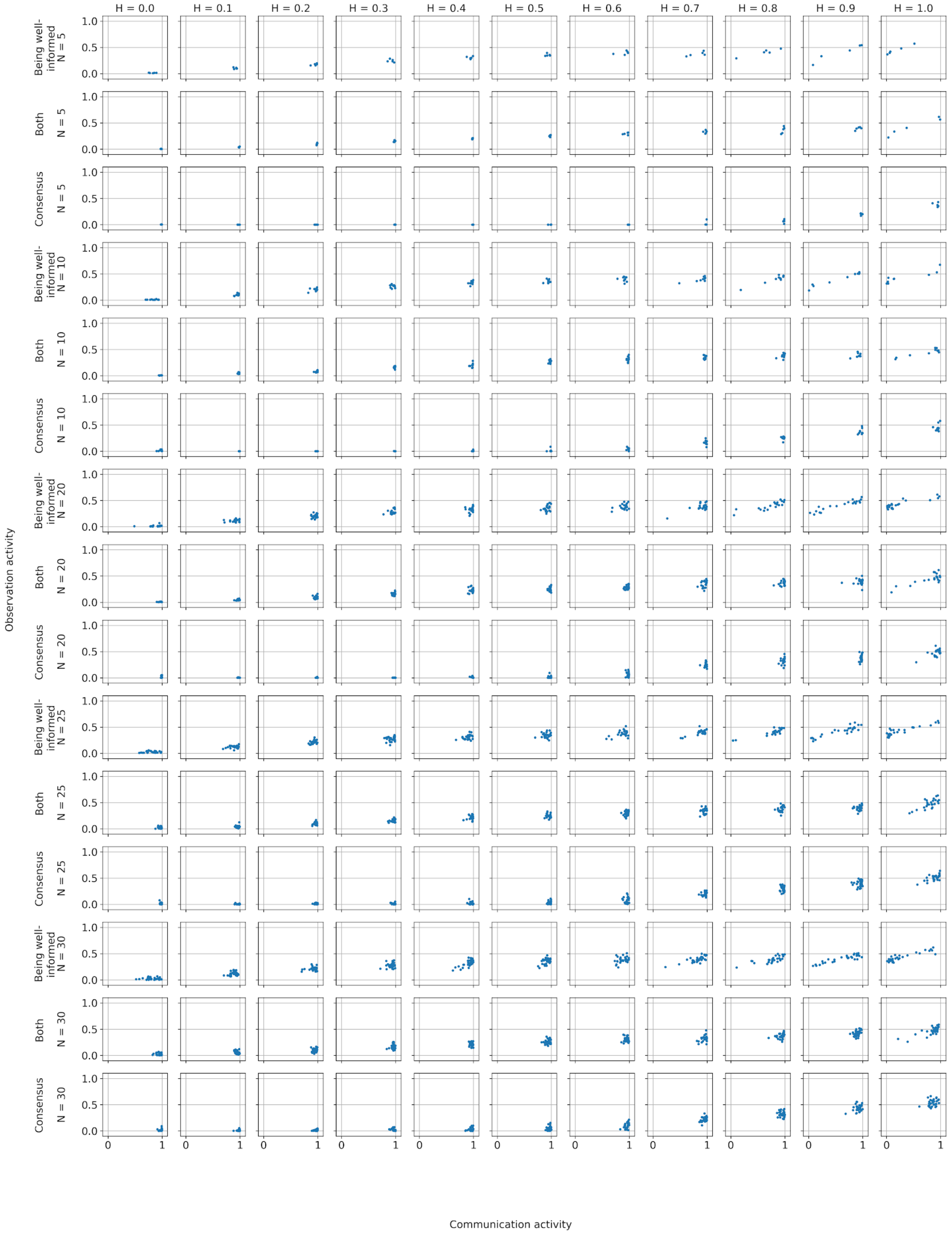

Figure 2.7. Average observation activities as a function of average communication activities, for $H \in \{0.0,\ 0.1,\ \ldots,\ 1.0\}$and $N\ \in \left\{5,\ 10,\ 20,\ 25,\ 30\ \right\}$parameters

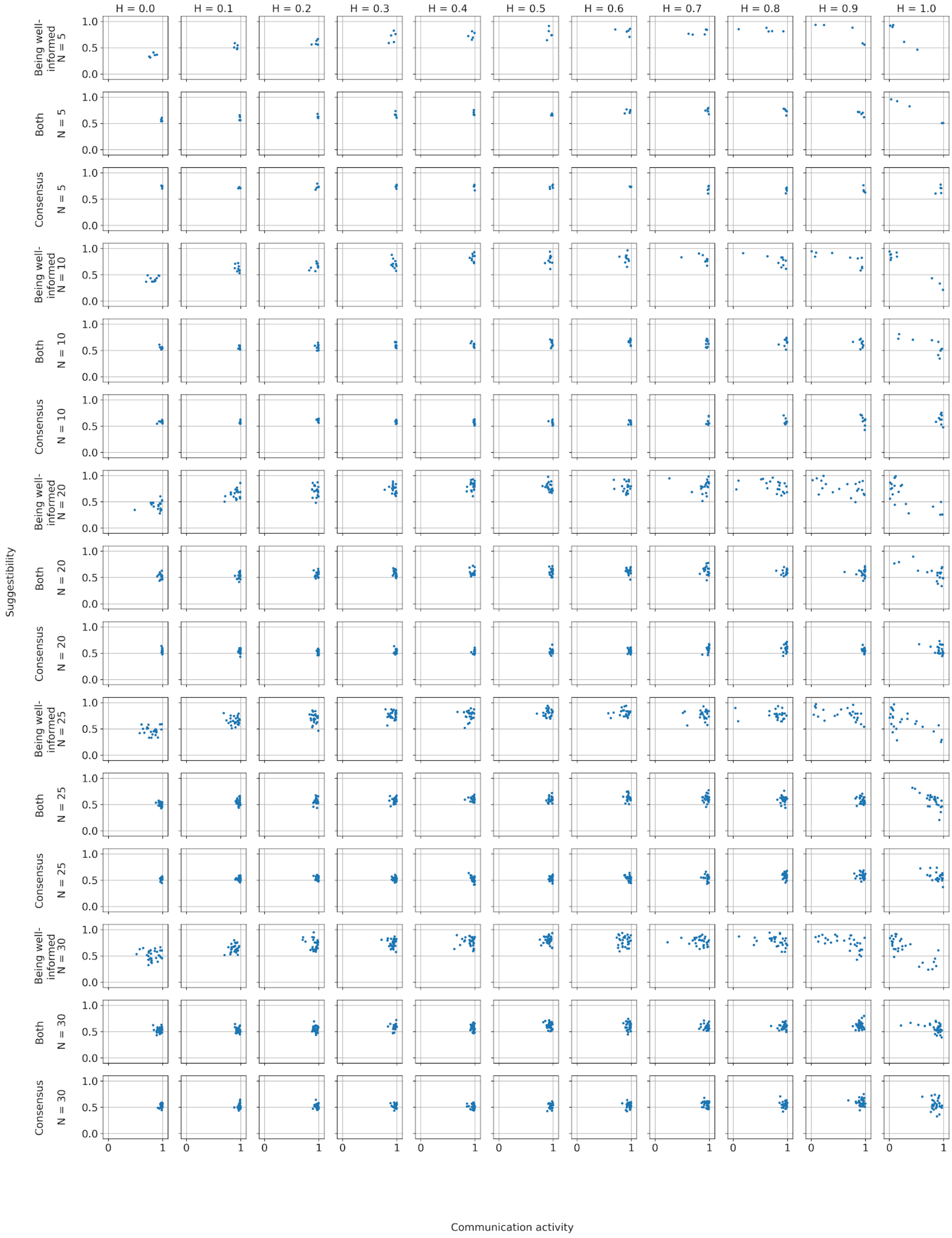

Figure 2.8. Average suggestibility levels as a function of average communication activities, for $H \in \{0.0,\ 0.1,\ \ldots,\ 1.0\}$and $N\ \in \left\{5,\ 10,\ 20,\ 25,\ 30\ \right\}$parameters

3. <u>Number of steps in the core function</u>

R marks the number of rounds in each run. The robustness of our model has also been investigated with values $R \in \{25, 50, 100\}$.

The full graph for groups aiming to achieve consensus is present regardless of the value of R: Figure 3.1 (3rd, 6th, 9th row) presents that the weighted in- and out-degrees of their communication networks are around 1 for all H, and the weighted outdegrees deviate very little (Figure 3.2). For R = 100, H = 1 the group becomes slightly more hierarchical and the outdegrees deviate relatively more than in other cases.

Hierarchical groups (Figure 3.3) and specialised agents (Figure 3.1) regarding the spread of information appear in groups that optimise on being well-informed for H>0.5, independently of R. For H=0 and R=100 there is a slight increase in the hierarchy, as there is a small decrease in the average communication activity too (Figure 3.4) (there is an inverse correlation between the hierarchy of the group and the average communication activity, for all parameters). For $0 < H \leq 0.5$ the communication network is full graph.

The optimal amount of average observation increases with the growth of H almost for all R (Figure 3.4). Groups that want to be well-informed have higher observation activity in general than the groups that want to achieve consensus. One exception is (when the group optimises on reaching consensus) at R = 25. 25 steps seem to be insufficient to observe a complex environment with K = 20 and observation activity does not raises at large H values. However, we investigated this case with a smaller K value (K = 5, Figure 3.7a) and we noticed that it produces similar results to our main statements. Thus, there is a connection between the complexity of environment and the "time" that is available to the agents to observe the environment and it is not worth to choose a too high K parameter for a relatively small R value. The communication activities behave similarly for all R value (although for R = 100 there is a small decrease in for H = 1, as mentioned before).

The H dependence of accuracy and consensus is also similar for different R values (Figure 3.5), however, the more "time" the group has, the more accurate they can get and the higher the level of their agreement is. Consensus aiming groups do not have enough time to reach high accuracy for high H within 25 rounds for an environment with K = 20 complexity, although this does not happen for a smaller K (K = 5, Figure 3.7b).

Suggestibility values are higher for groups aiming to be well-informed for H>0, independently of R, and lower for consensus aiming groups (Figure 3.6). With the decrease of R, there is a decrease in suggestibility levels too. For R = 25, this phenomenon is less emphasised with K = 5 (Figure 3.7c).

Figure 3.8 and 3.9 shows that in groups that aim to be well-informed at high access to information, the agents specialised on spreading the information are more active in observation too, independently of R, and also less suggestible.

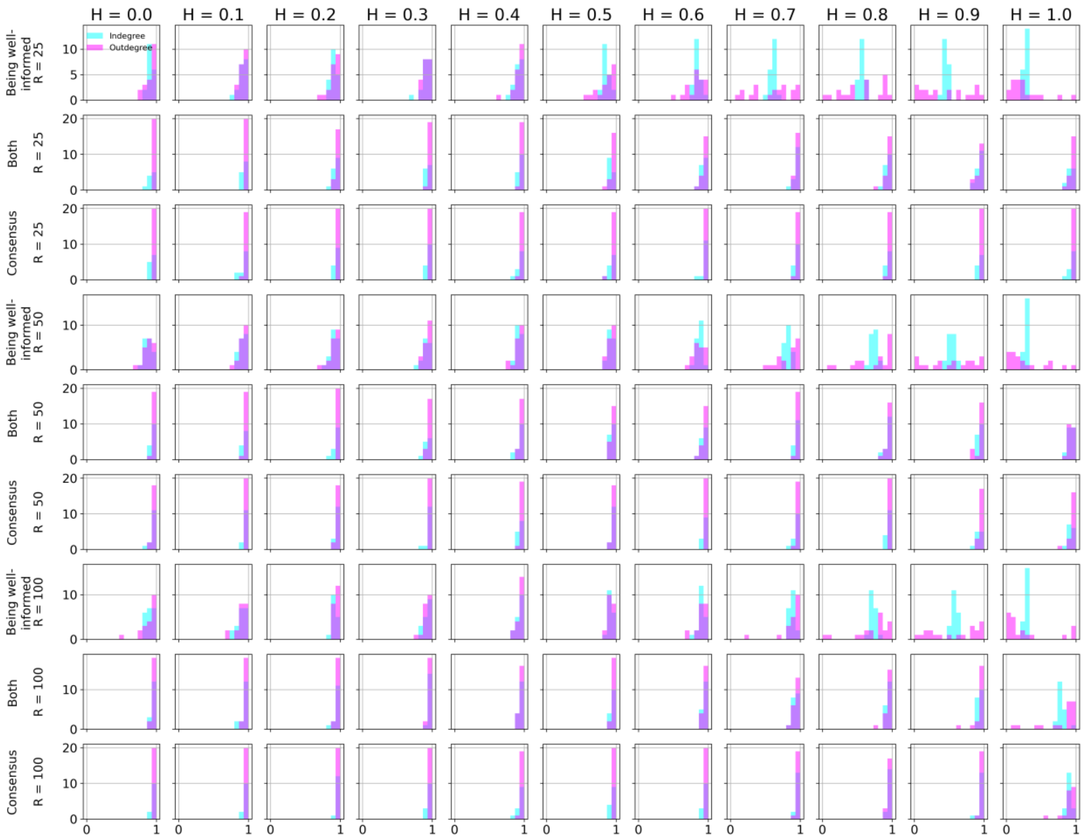

Figure 3.1. Histograms of the weighted in- and outdegrees for $H \in \{0.0,\ 0.1,\ \ldots,\ 1.0\}$ and $R \in \left\{25,\ 50,\ 100\right\}$ parameters

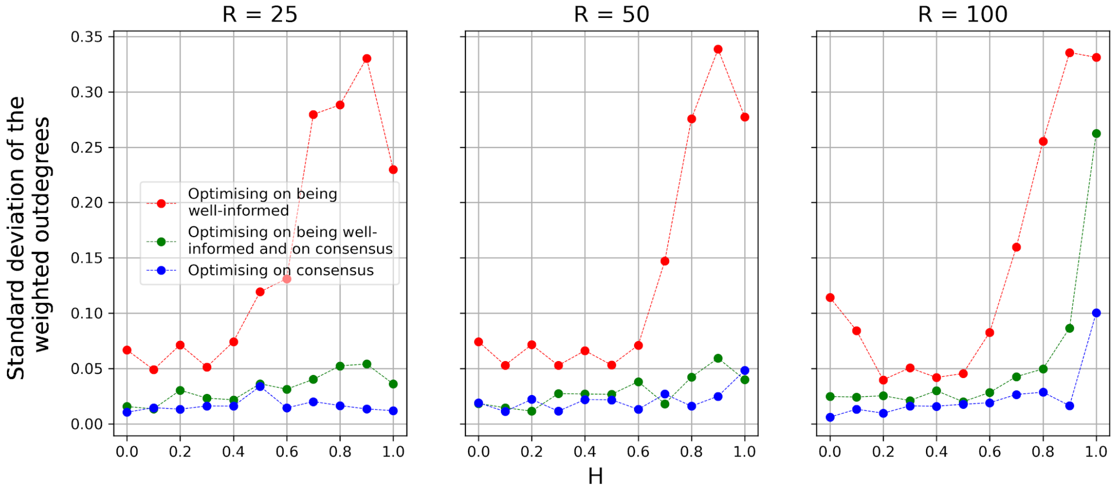

Figure 3.2. Standard deviation of the weighted outdegrees as a function of H, for $R \in \left\{25,\ 50,\ 100\right\}$ parameters

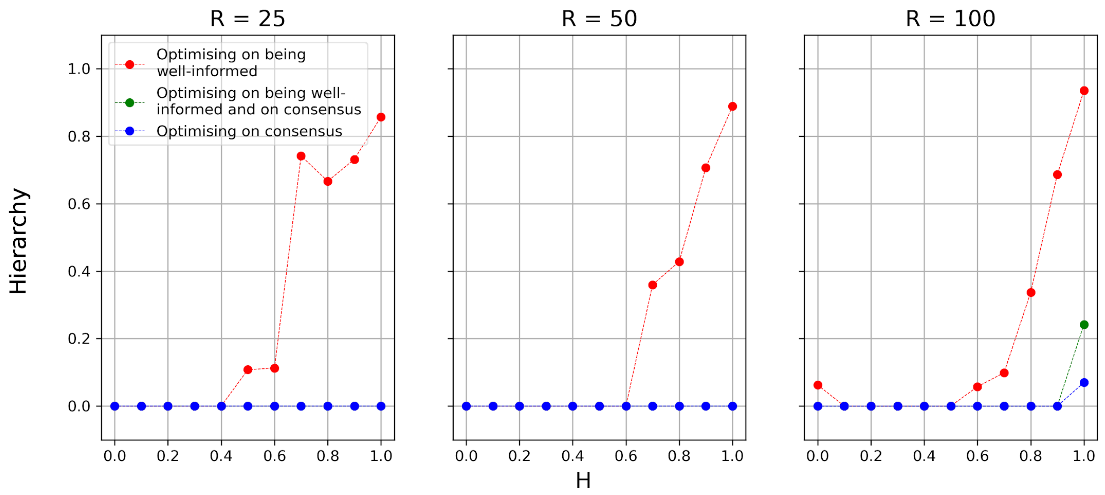

Figure 3.3. Hierarchy of the communication networks as a function of H, for $R \in \{25, 50, 100\}$

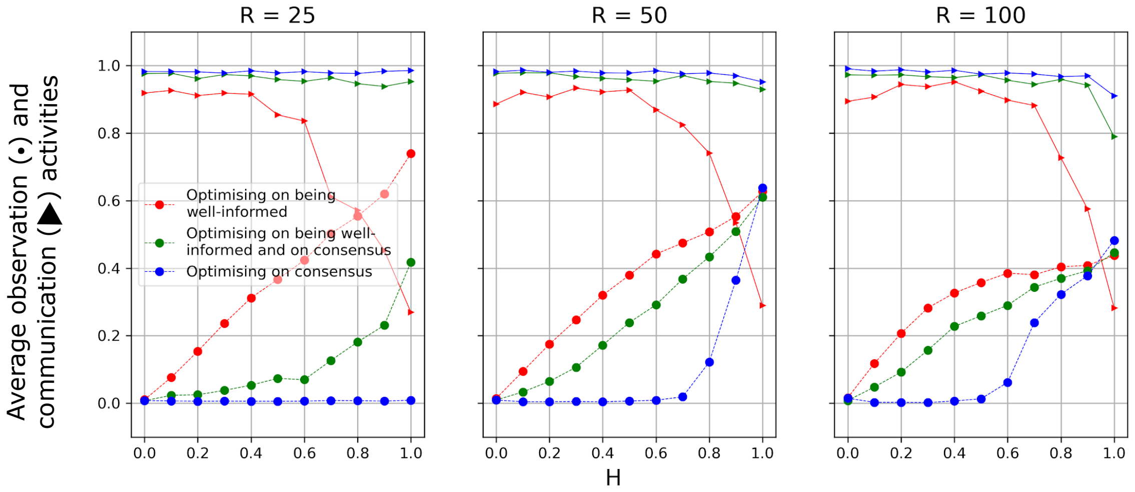

Figure 3.4. Average observation and average communication activities as a function of H, for $R \in \{25, 50, 100\}$ parameters

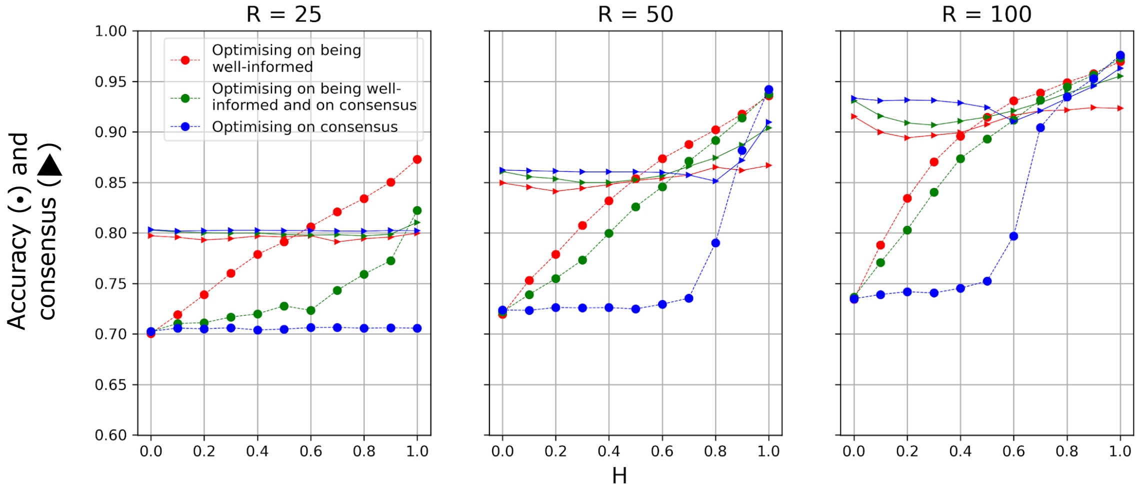

Figure 3.5. The level of accuracy and consensus as a function of H, for $R \in \{25, 50, 100\}$

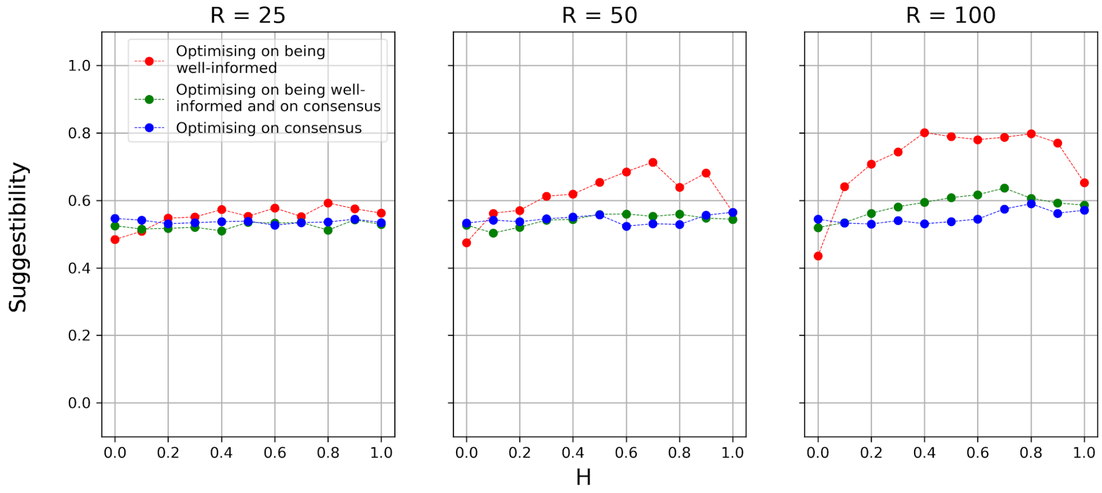

Figure 3.6. The average level of suggestibility of agents as a function of H, for $R \in \{25, 50, 100\}$

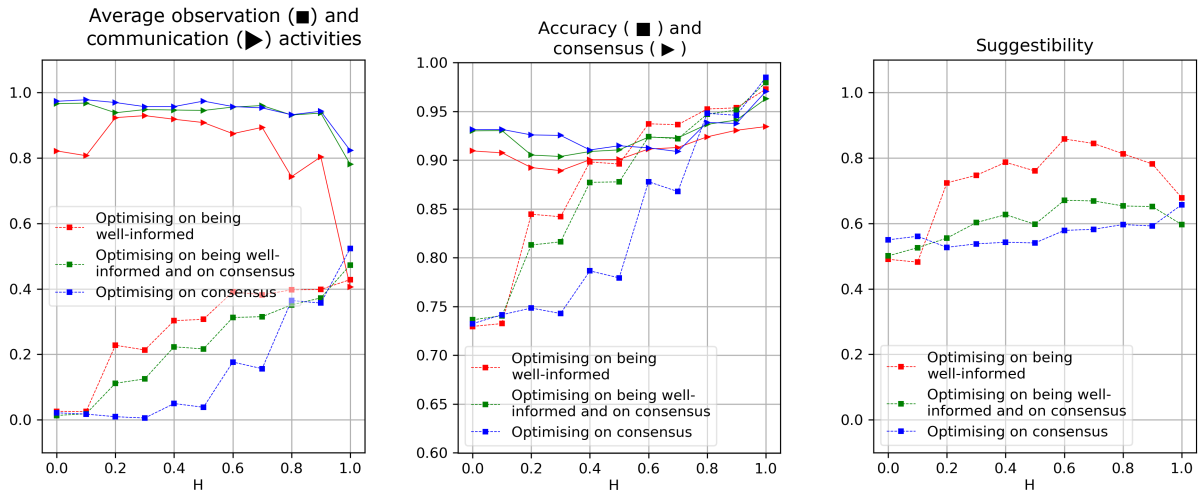

a b c

Figure 3.7 a. The average observation and communication activities as a function of H. b. The accuracy and consensus level of groups as a function of H. c. The average suggestibility levels as a function of H. All in case of R = 25, K = 5.

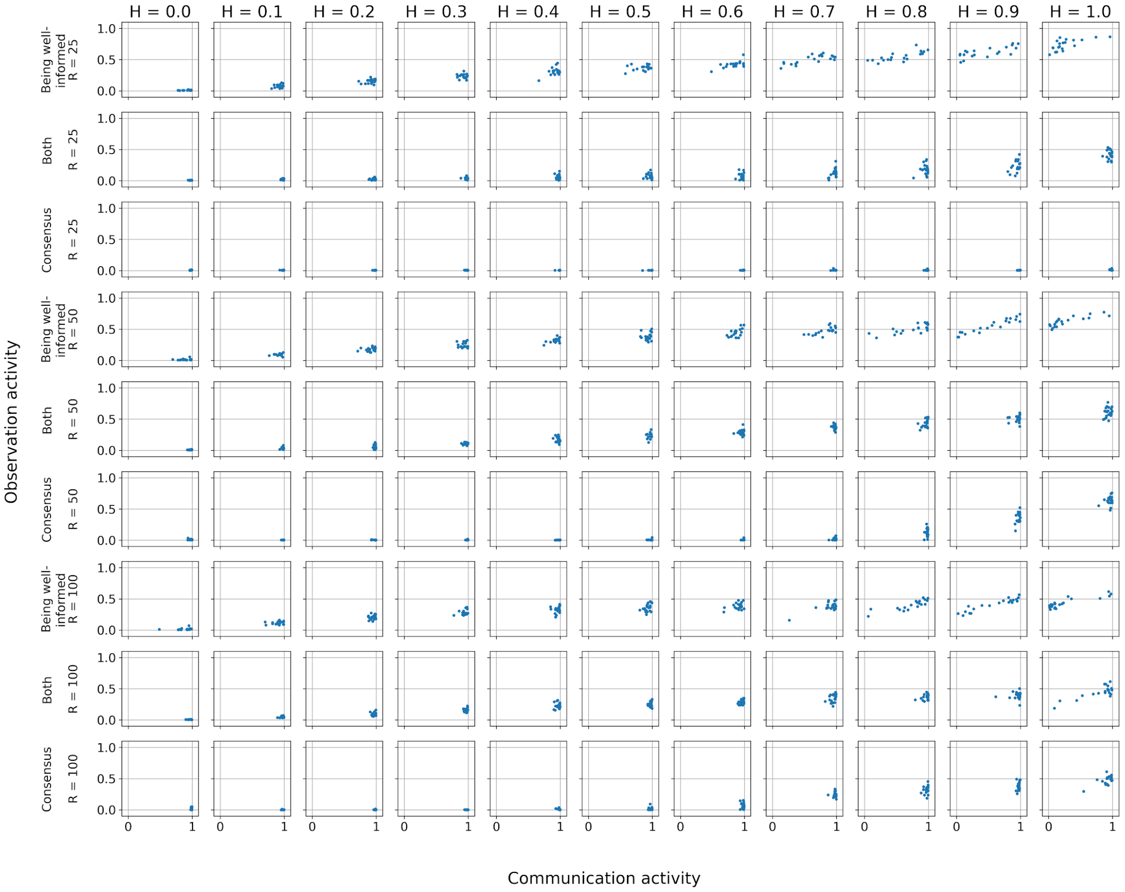

Figure 3.8. Average observation activities as a function of average communication activities for $H \in \{0.0, 0.1, \ldots, 1.0\}$ and $R \in \{25, 50, 100\}$ parameters

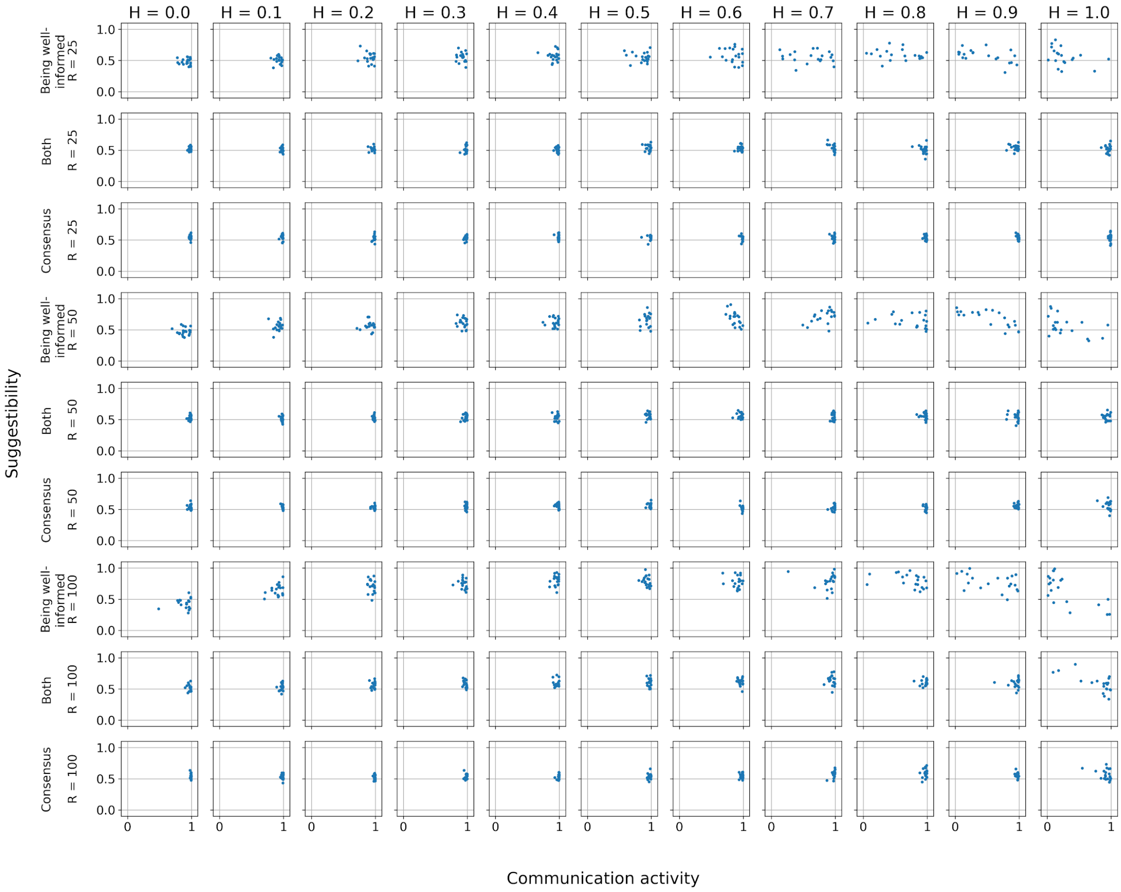

Figure 3.9. Suggestibility as a function of average communication activities for $H \in \{0.0,\ 0.1,\ \ldots,\ 1.0\}$and $R\ \in \left\{25,\ 50,\ 100\right\}$ parameters

4. <u>The cost of activities</u>

We have run simulations with multiple communication cost ($\kappa^{Comm}\ \in\ \{0, 0.05,\ 0.2\}$) and observation cost values ($\kappa^{Obs}\ \in\ \{0.3,\ 0.5,\ 0.8,\ 1.0\}$). According to our interpretation, observation is a much more costly activity than communication. When this relation is fulfilled, we can see on the figures below that our main results are valid and the tendency is repeated with different combinations of costs.

However, when there is no cost for communication, the networks are less hierarchical (Figure 4.5) since there is no need for optimising the structure of information flow and accordingly, the standard deviation of the outdegrees of these networks is lower too (Figure 4.4). Although, this is unrealistic because communication needs time and energy as well even if it is less than in the case of observation.

Also, when the cost of communication is relatively close to the cost of observation (e.g. κ^{Comm}= 0.2), the results are different. For example, at high H values, the networks of groups that want to reach consensus fall apart (because it no longer worths to communicate, Figure 4.5,

illustrated with negative hierarchy values) and this is emphasised by the peaks at 0 on the in- and out-degree histograms (Figures 4.1, 4.2, 4.3) and by the average communication activity curves (Figure 4.6) . The high deviation in hierarchy for κ^{Comm}= 0.2 is due to the small size of the largest connected components (which are considerably smaller than N). Also, the increase of observation cost slightly deteriorates the overall accuracy of groups (Figure 4.7).

Higher suggestibility values are correlated with groups that want to be well-informed for all simulations indifferently of costs (Figure 4.8). In groups where hierarchy emerges, the agents who communicate more, have higher observation activities and lower suggestibility levels (Figures 4.9 - 4.14).

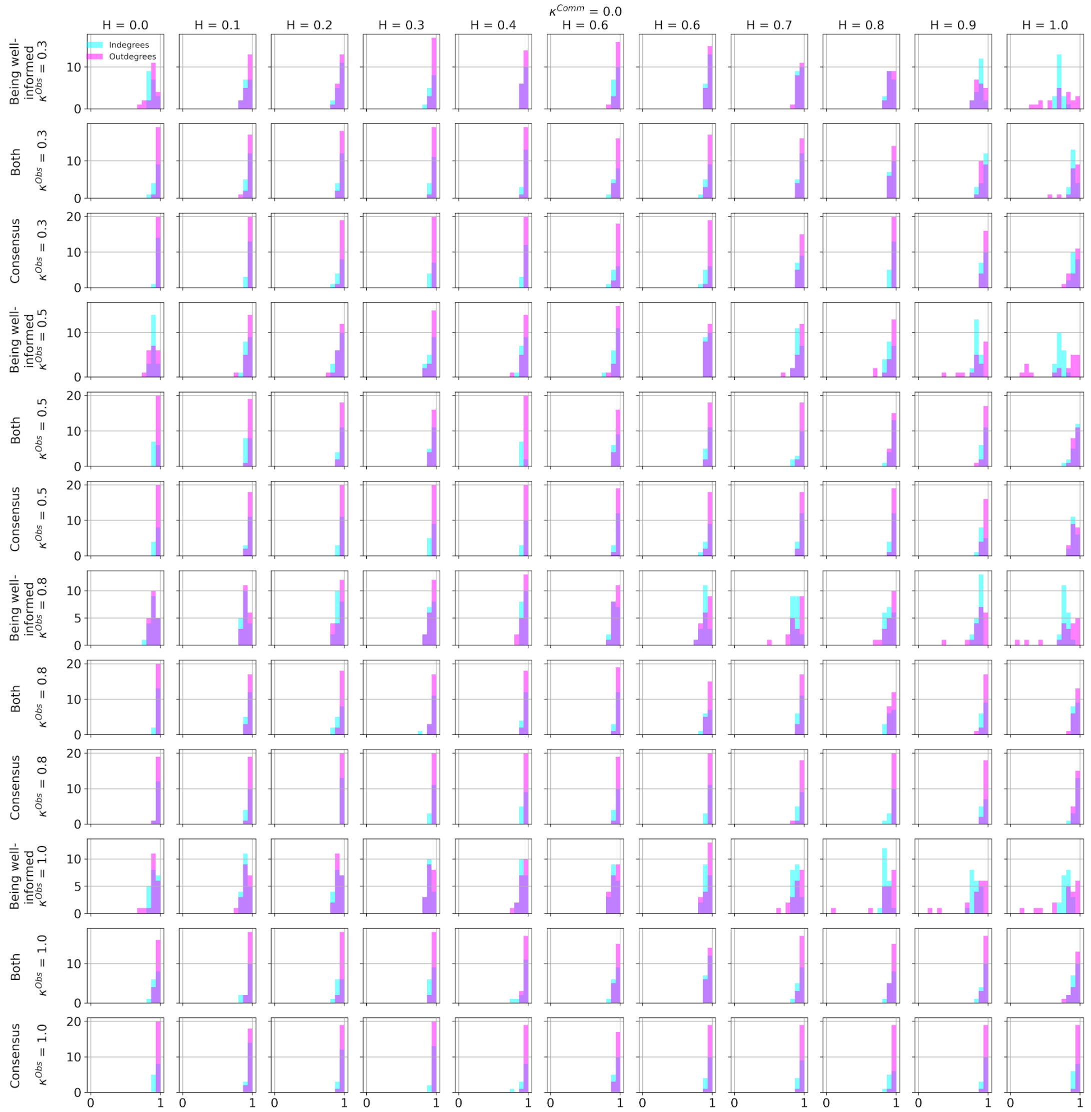

Figure 4.1. Histograms of the weighted in- and outdegrees for $H \in \{0.0,\ 0.1,\ \ldots,\ 1.0\}$, $\kappa^{Obs} \in \{0.3,\ 0.5,\ 0.8,\ 1.0\}$and $\kappa^{Comm} = 0$

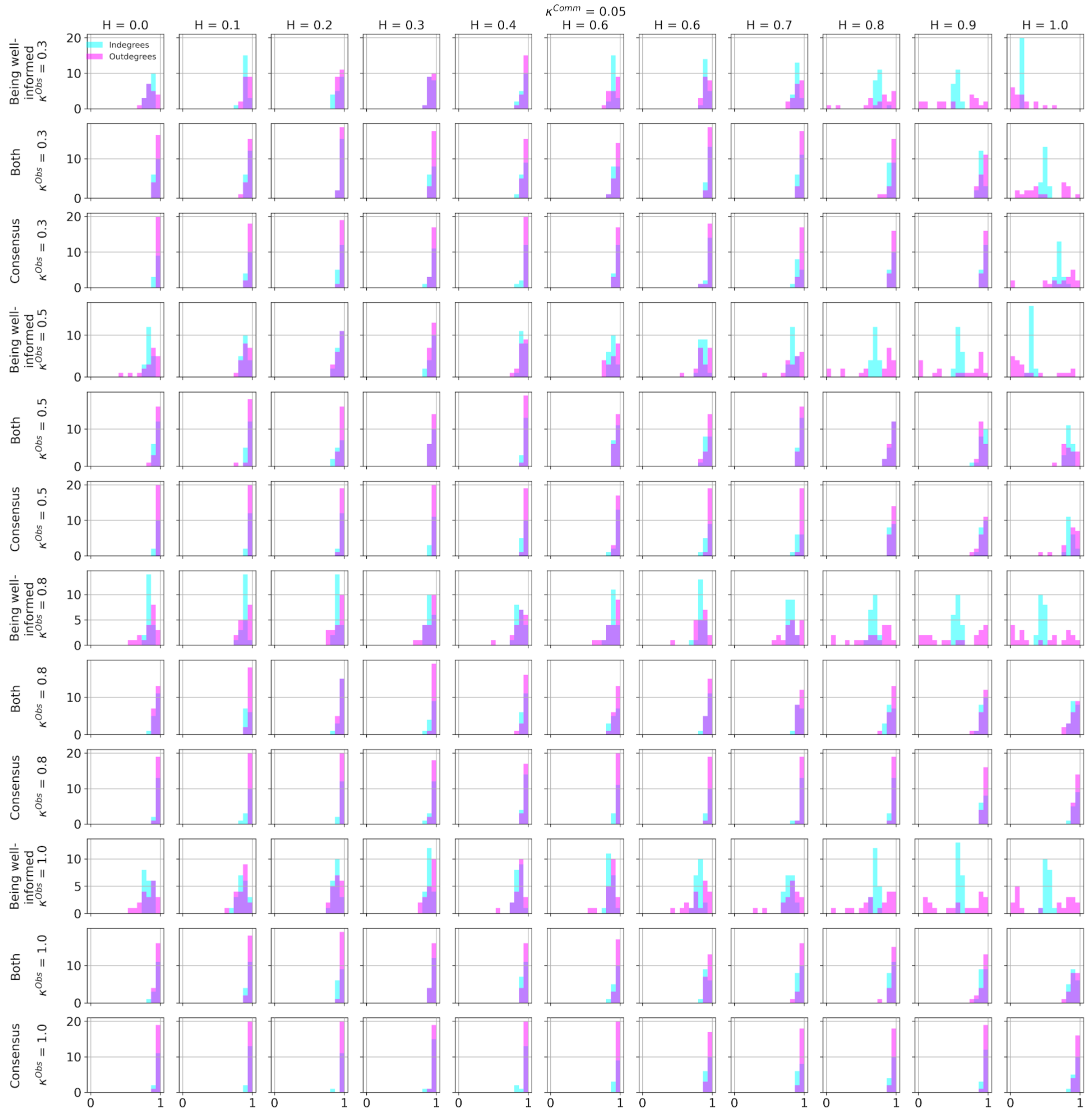

Figure 4.2. Histograms of the weighted in- and outdegrees for $H \in \{0.0,\ 0.1,\ \ldots,\ 1.0\}$, $\kappa^{Obs} \in \{0.3,\ 0.5,\ 0.8,\ 1.0\}$and $\kappa^{Comm} = 0.05$

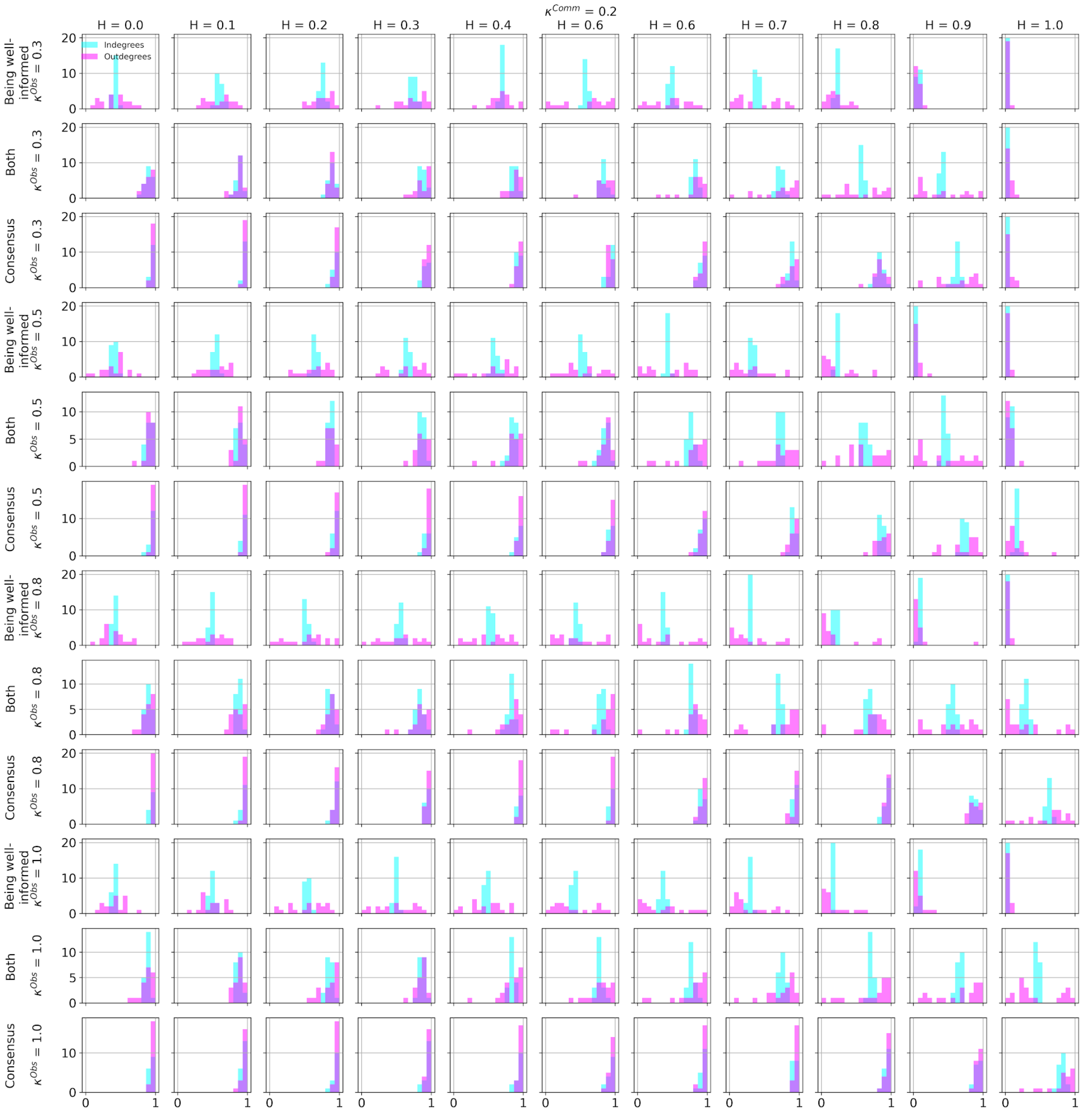

Figure 4.3. Histograms of the weighted in- and outdegrees for $H \in \{0.0,\ 0.1,\ \ldots,\ 1.0\}$, $\kappa^{Obs} \in \{0.3,\ 0.5,\ 0.8,\ 1.0\}$and $\kappa^{Comm} = 0.2$

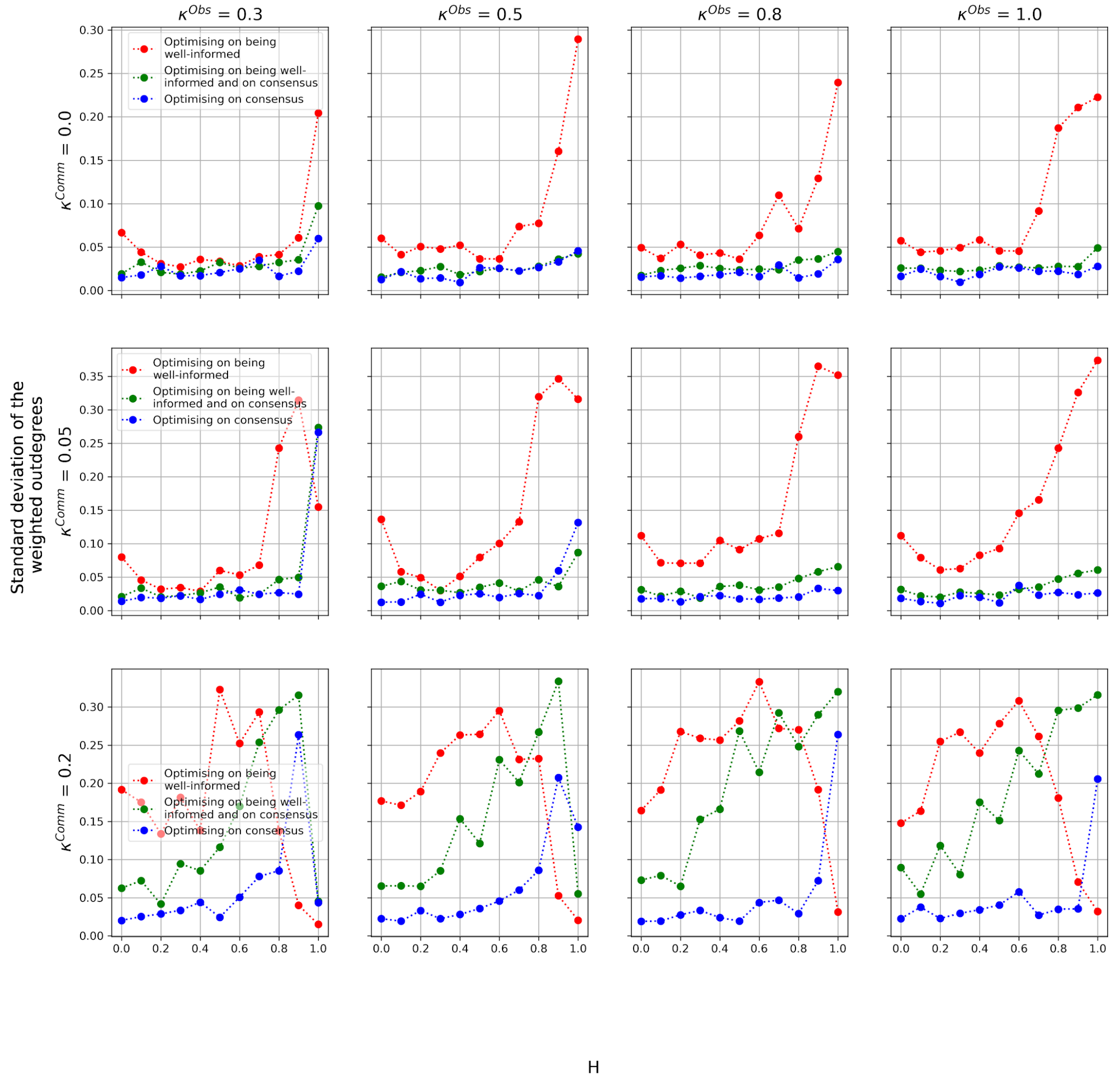

Figure 4.4. Standard deviation of the weighted outdegrees as a function of H, for $\kappa^{Comm} \in \{0, 0.05,\ 0.2\}$and $\kappa^{Obs} \in \{0.3,\ 0.5,\ 0.8,\ 1.0\}$parameters

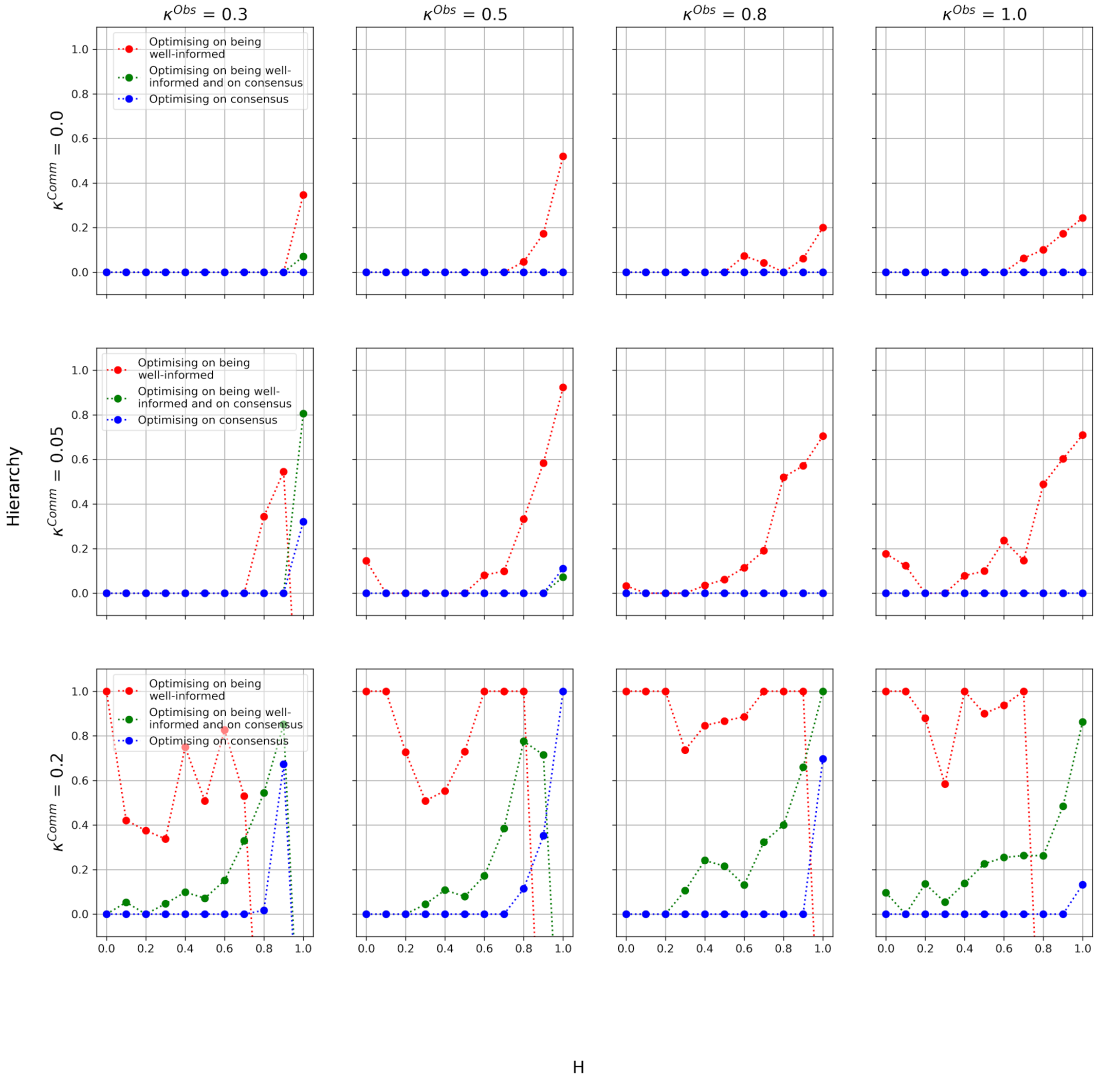

Figure 4.5. Hierarchy of the communication networks as a function of H, for $\kappa^{Comm} \in \{0, 0.05, 0.2\}$and $\kappa^{Obs} \in \{0.3, 0.5, 0.8, 1.0\}$parameters

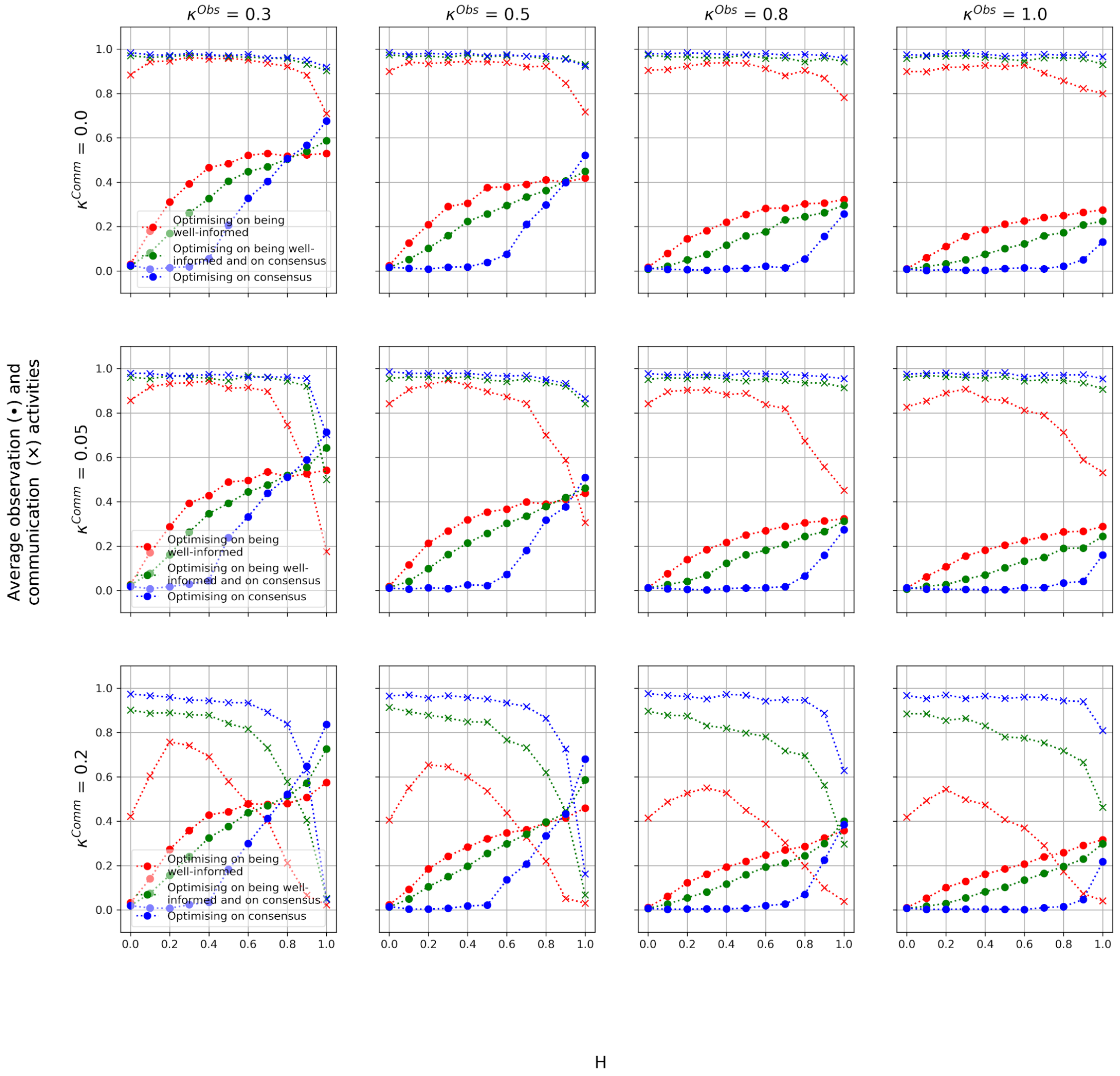

Figure 4.6. Average observation and average communication activities as a function of H, for $\kappa^{Comm} \in \{0, 0.05, 0.2\}$and $\kappa^{Obs} \in \{0.3, 0.5, 0.8, 1.0\}$parameters

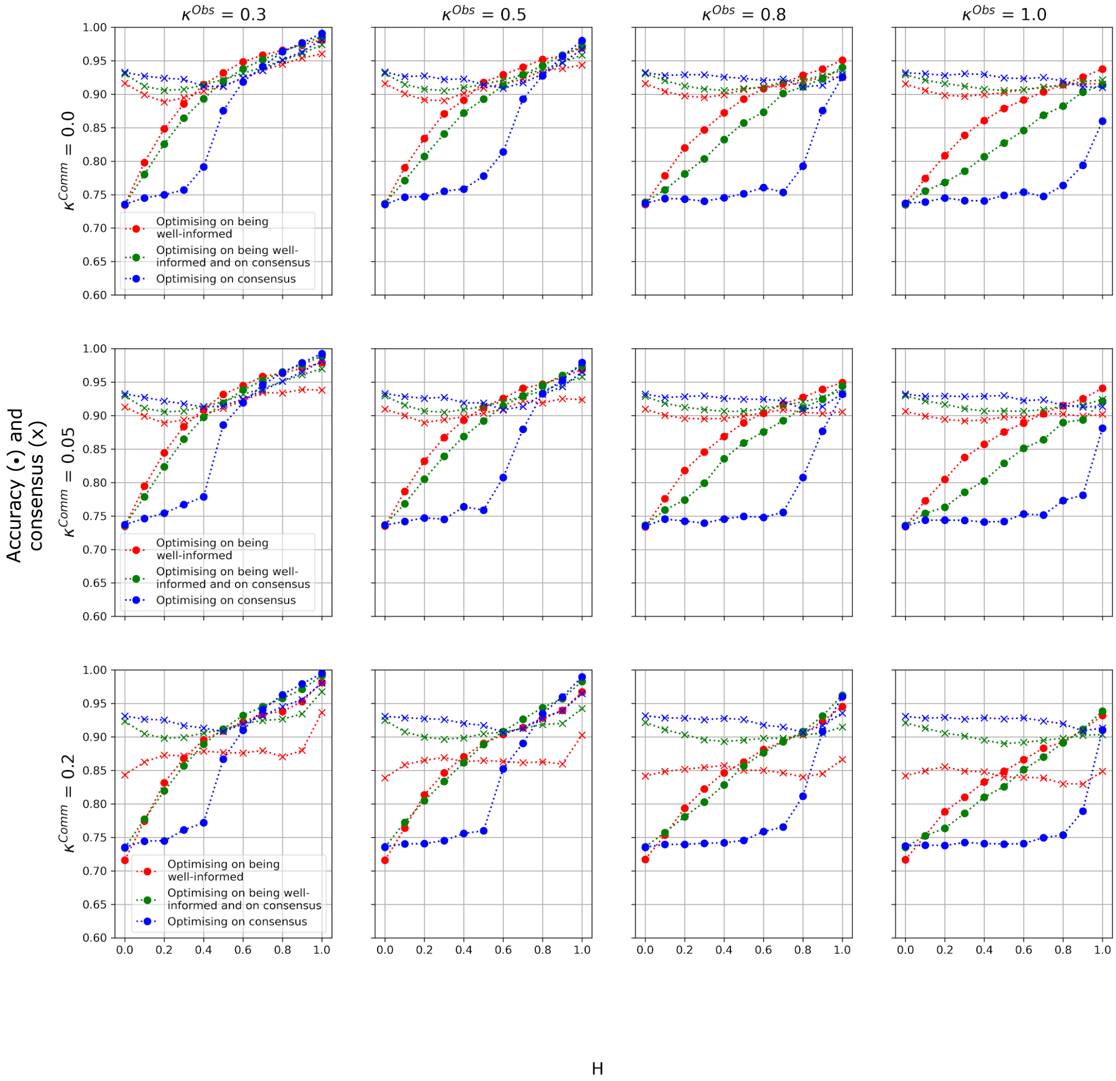

Figure 4.7. The level of accuracy and consensus as a function of H, for $\kappa^{Comm} \in \{0, 0.05, 0.2\}$ and $\kappa^{Obs} \in \{0.3, 0.5, 0.8, 1.0\}$parameters

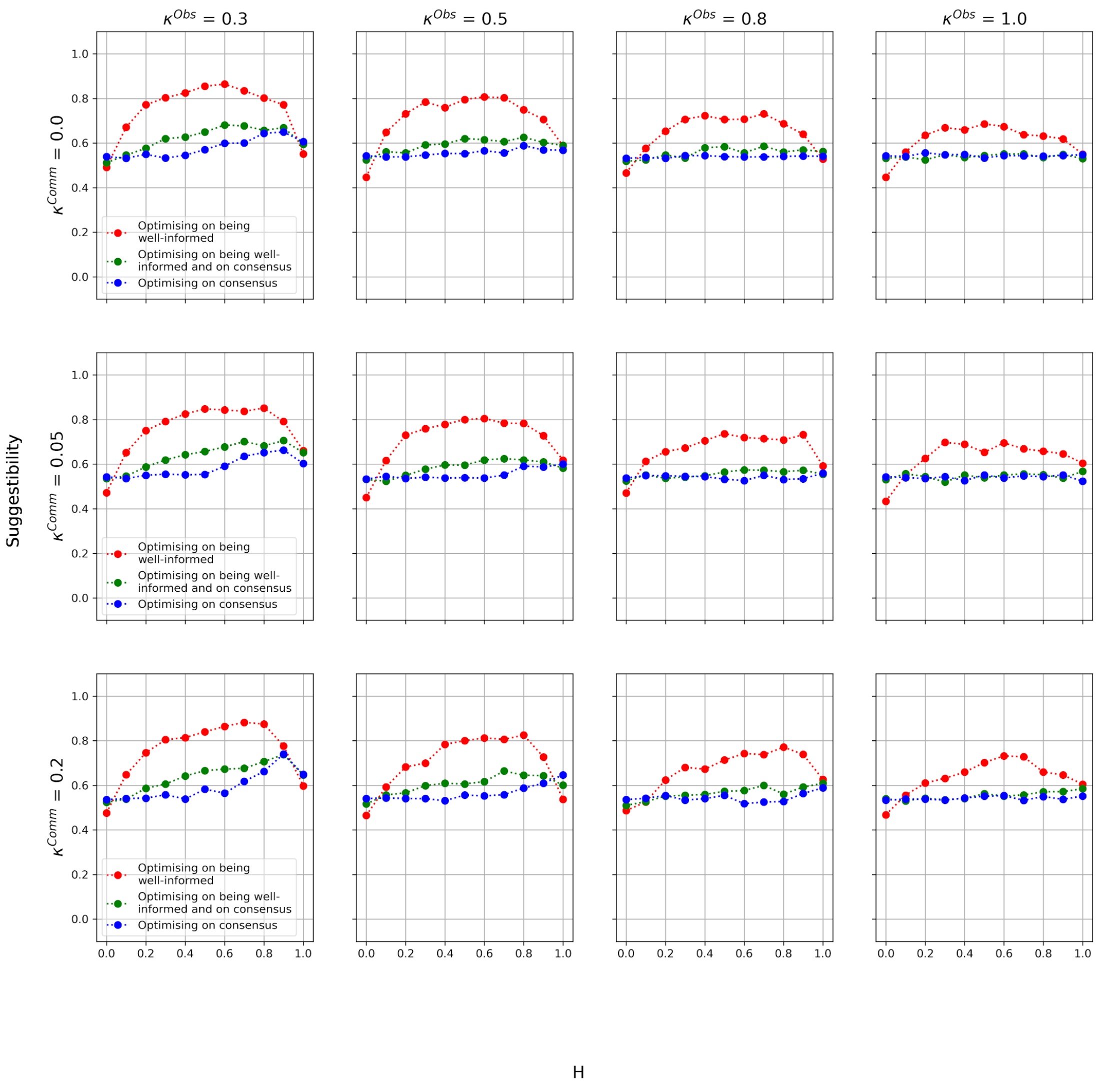

Figure 4.8. The average level of suggestibility of agents as a function of H, for $\kappa^{Comm} \in \{0, 0.05, 0.2\}$and $\kappa^{Obs} \in \{0.3, 0.5, 0.8, 1.0\}$parameters

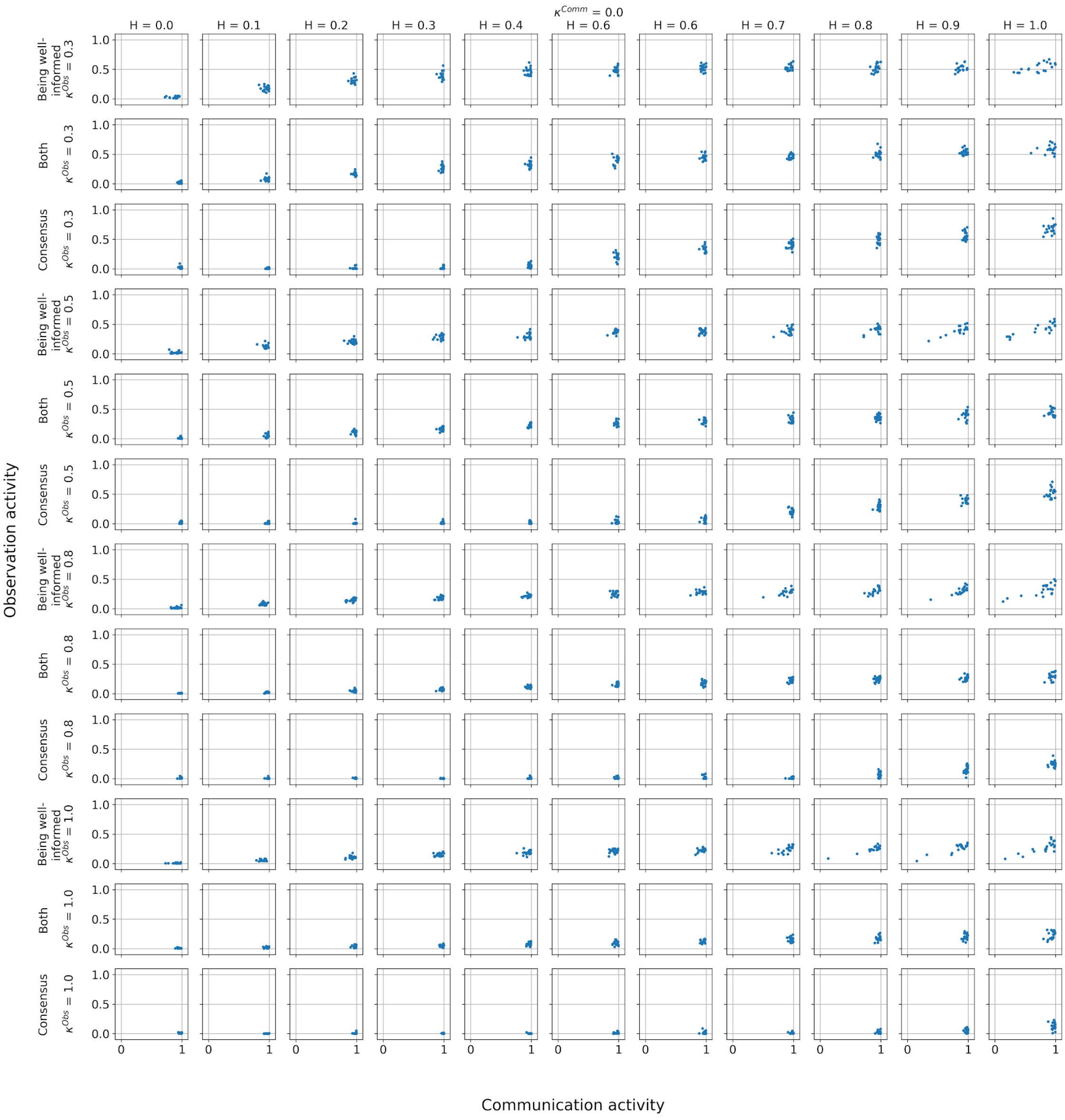

Figure 4.9. Average observation activities as a function of average communication activities for $H \in \{0.0,\ 0.1,\ \ldots,\ 1.0\}$, $\kappa^{Comm} = 0$ and $\kappa^{Obs} \in \{0.3,\ 0.5,\ 0.8,\ 1.0\}$parameters

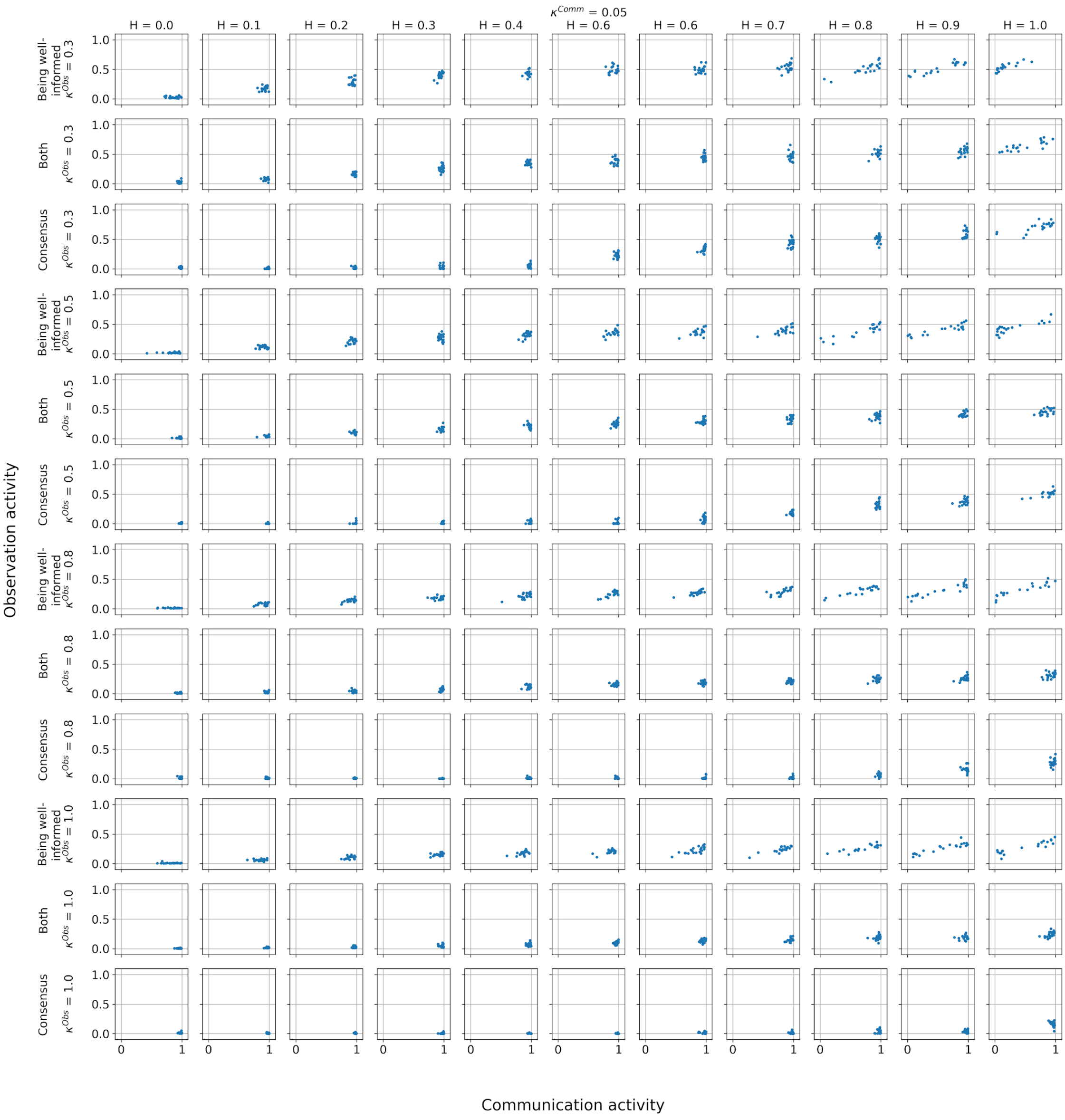

Figure 4.10. Average observation activities as a function of average communication activities for $H \in \{0.0,\ 0.1,\ \ldots,\ 1.0\}$, $\kappa^{Comm}\ = 0.05$and $\kappa^{Obs}\ \in\ \{0.3,\ 0.5,\ 0.8,\ 1.0\}$parameters

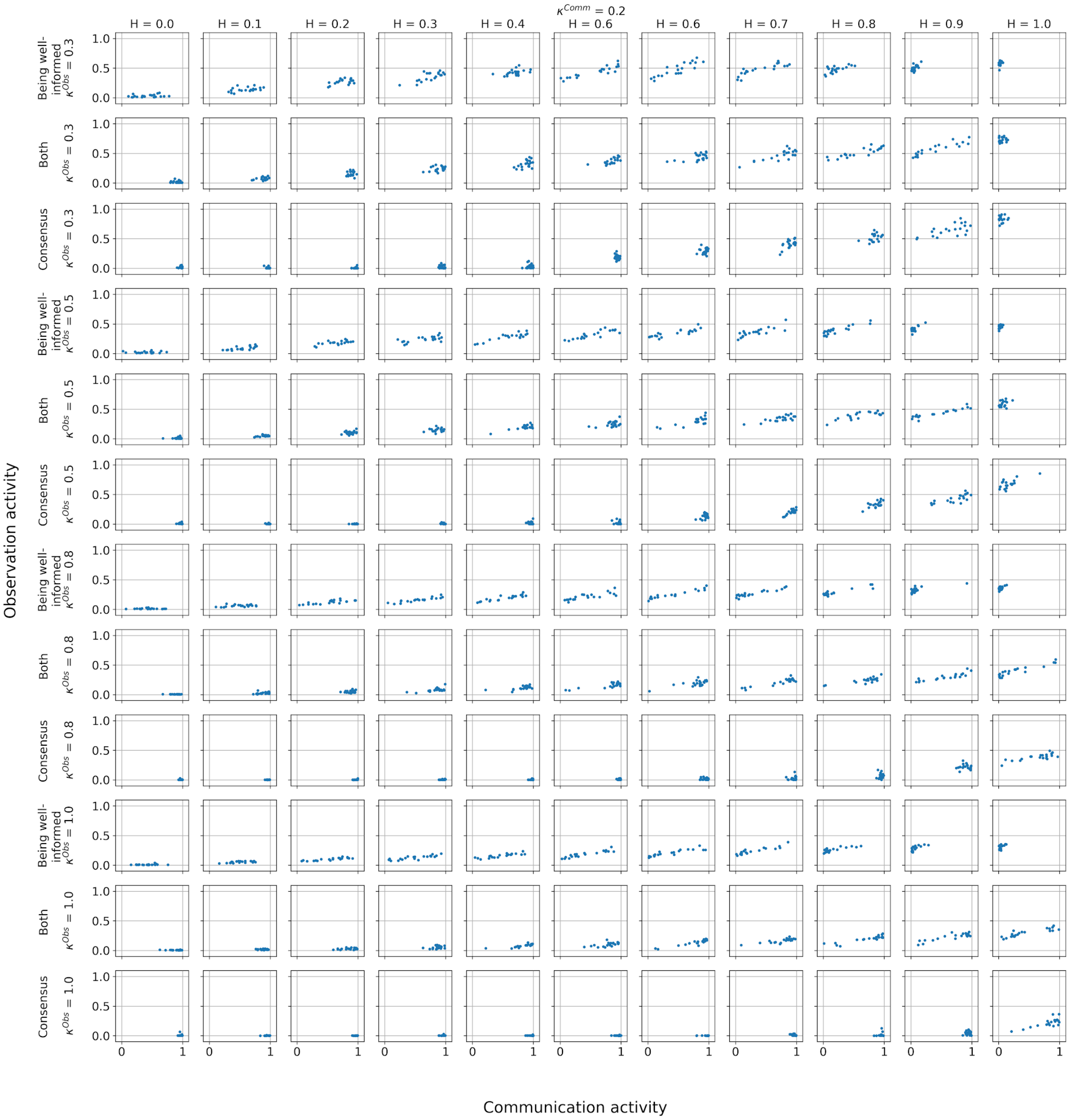

Figure 4.11. Average observation activities as a function of average communication activities for $H \in \{0.0,\ 0.1,\ \ldots,\ 1.0\}$, $\kappa^{Comm} = 0.2$and $\kappa^{Obs} \in \{0.3,\ 0.5,\ 0.8,\ 1.0\}$parameters

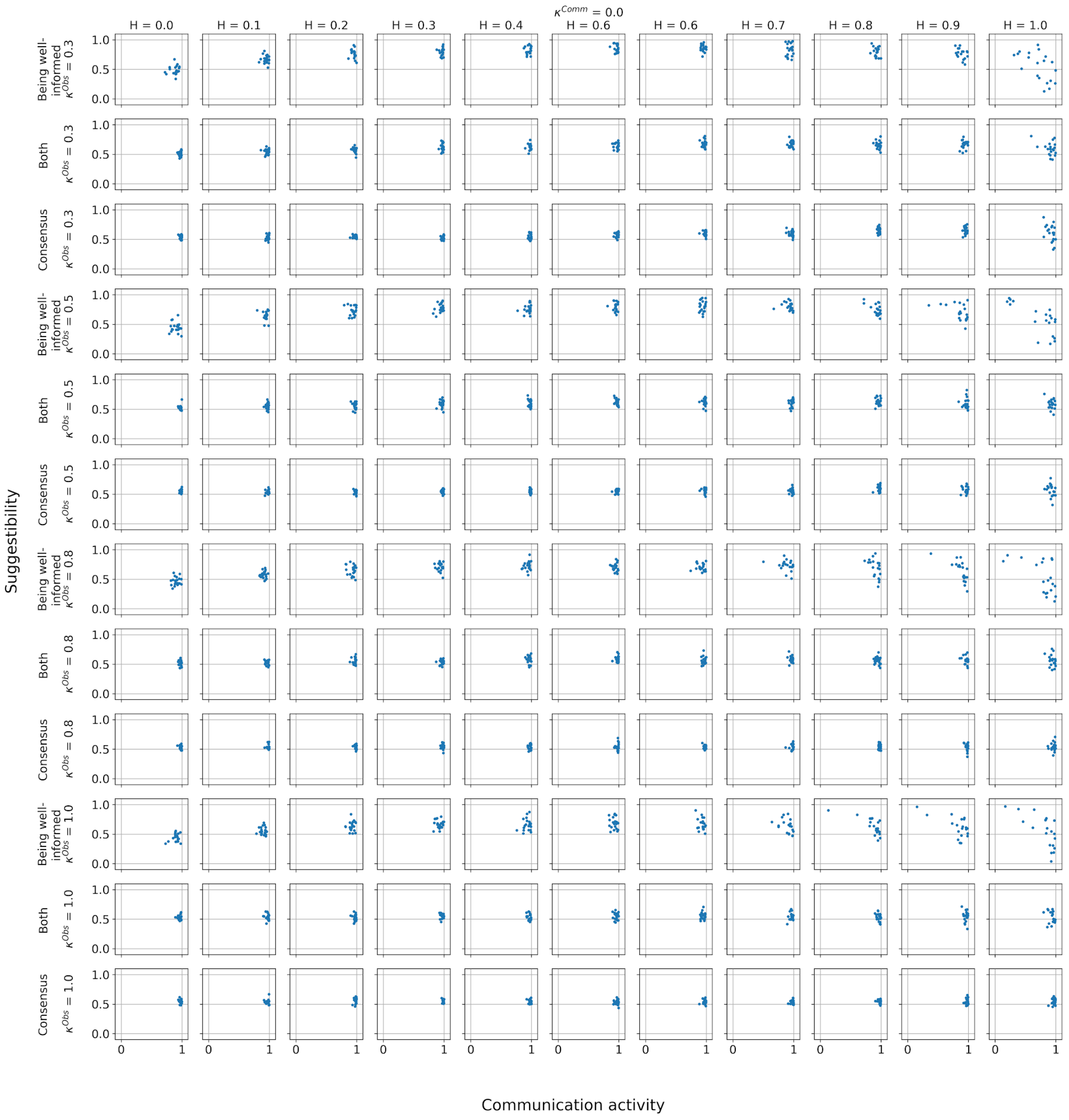

Figure 4.12. Suggestibility as a function of average communication activities for $H \in \{0.0,\ 0.1,\ \ldots,\ 1.0\}$, $\kappa^{Comm} = 0$and $\kappa^{Obs} \in \{0.3,\ 0.5,\ 0.8,\ 1.0\}$parameters

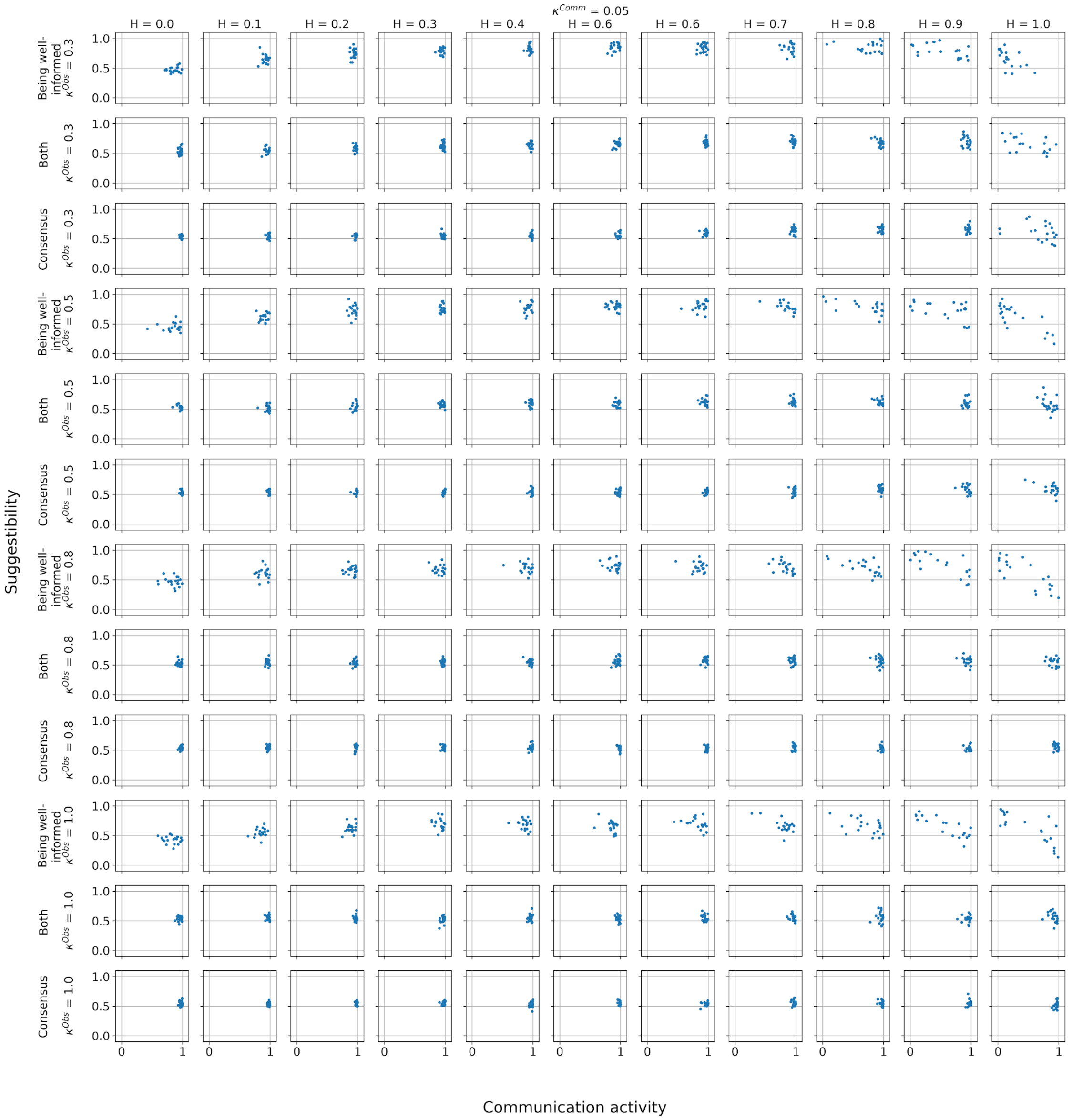

Figure 4.13. Suggestibility as a function of average communication activities for $H \in \{0.0,\ 0.1,\ \ldots,\ 1.0\}$, $\kappa^{Comm} = 0.05$ and $\kappa^{Obs} \in \{0.3,\ 0.5,\ 0.8,\ 1.0\}$parameters

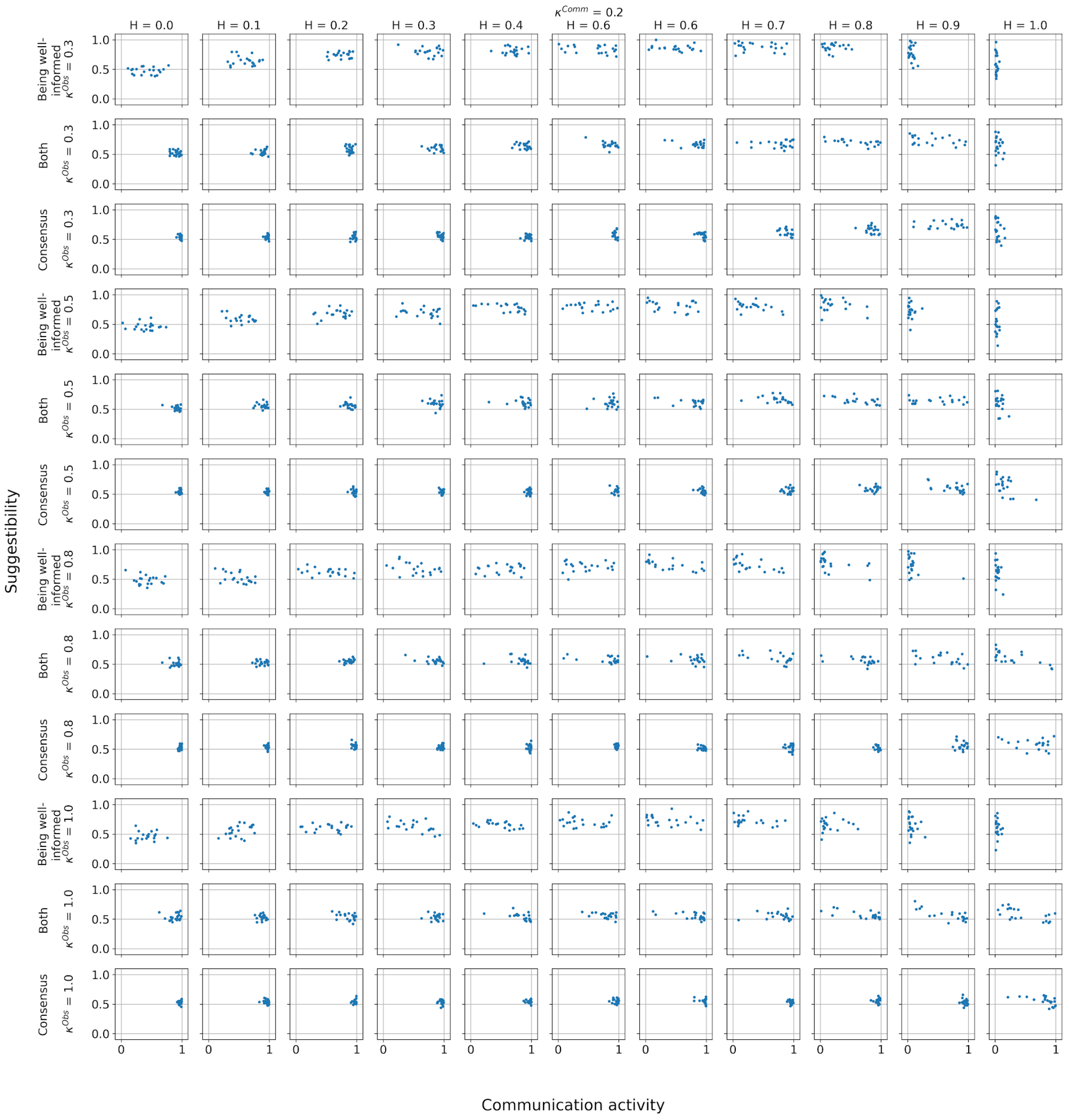

Figure 4.14. Suggestibility as a function of average communication activities for $H \in \{0.0,\ 0.1,\ \dots,\ 1.0\}$, $\kappa^{Comm} = 0.2$ and $\kappa^{Obs} \in \{0.3,\ 0.5,\ 0.8,\ 1.0\}$ parameters

In summary, we proposed a model to study the features of optimal decision making groups. We found that, independently of the main parameters of the simulation - K, N, R, L -, our main conclusion are valid. In addition, while the main statements remain true, we have discovered some further insights into the nature of group decision making during the parameter sweep - such as the correlation between the level of consensus and "time" (number of steps, R) that is available to yield information about the environment, between accuracy and “time", and the relationship between the R parameter and the complexity of the environment. When choosing the parameter intervals, we considered both their relevance and our computational capacity. While one of our two assumptions

was that the cost of communication is essentially lower than the observation cost, unsurprisingly the communication network falls apart for high communication cost and high H values. Lastly, our results - and their general nature, regardless of parameters - might be a proof of raison d’être of the model introduced in this article.
